# Supplementary material for: Food-based multisensory stimulation ameliorates cognitive impairment after mild traumatic brain injury in male rats by modulating intestinal and brain inflammation
Source: PLoS One. 2026 Feb 27;21(2):e0343814. doi: 10.1371/journal.pone.0343814 (PMC12948070; doi:10.1371/journal.pone.0343814)
Supplement: S1 File — (PDF) [file pone.0343814.s001.pdf]

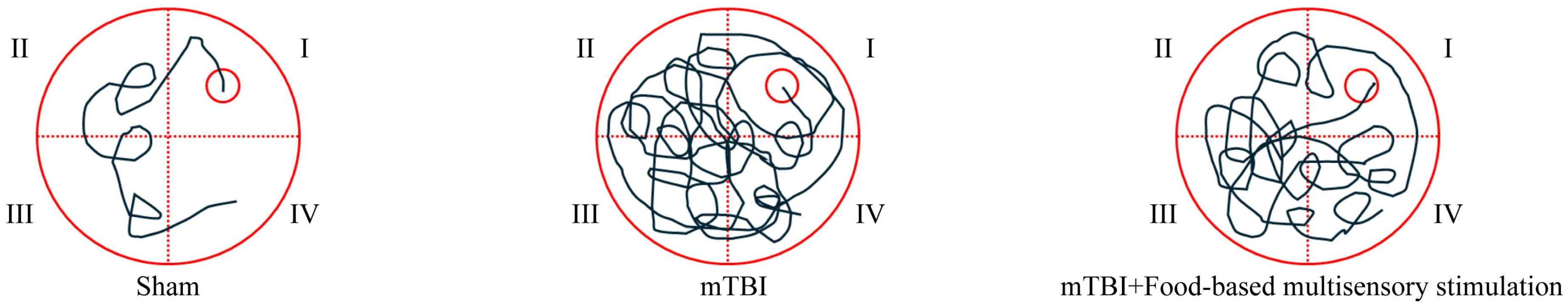

II
I
III
IV
Sham
II
I
III
IV
mTBI
II
I
III
IV
mTBI+Food-based multisensory stimulation

Fig 2 A

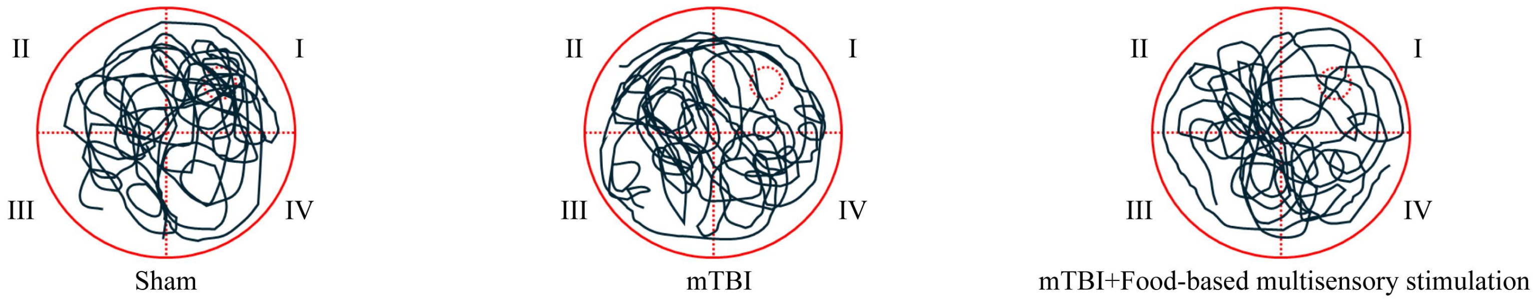
II
I
III
IV
Sham
II
I
III
IV
mTBI
II
I
III
IV
mTBI+Food-based multisensory stimulation

Fig 2 B

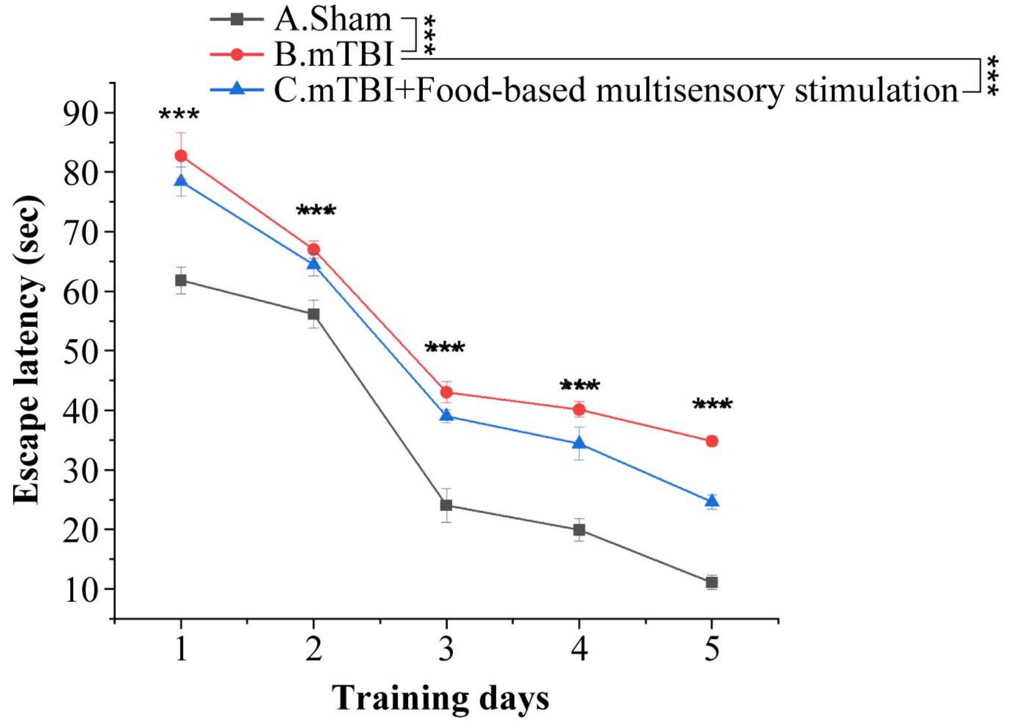
A.Sham
B.mTBI
C.mTBI+Food-based multisensory stimulation
***
***
Escape latency (sec)
90
80
70
60
50
40
30
20
10
***
***
***
***
***
1
2
3
4
5
Training days

Fig 2 C

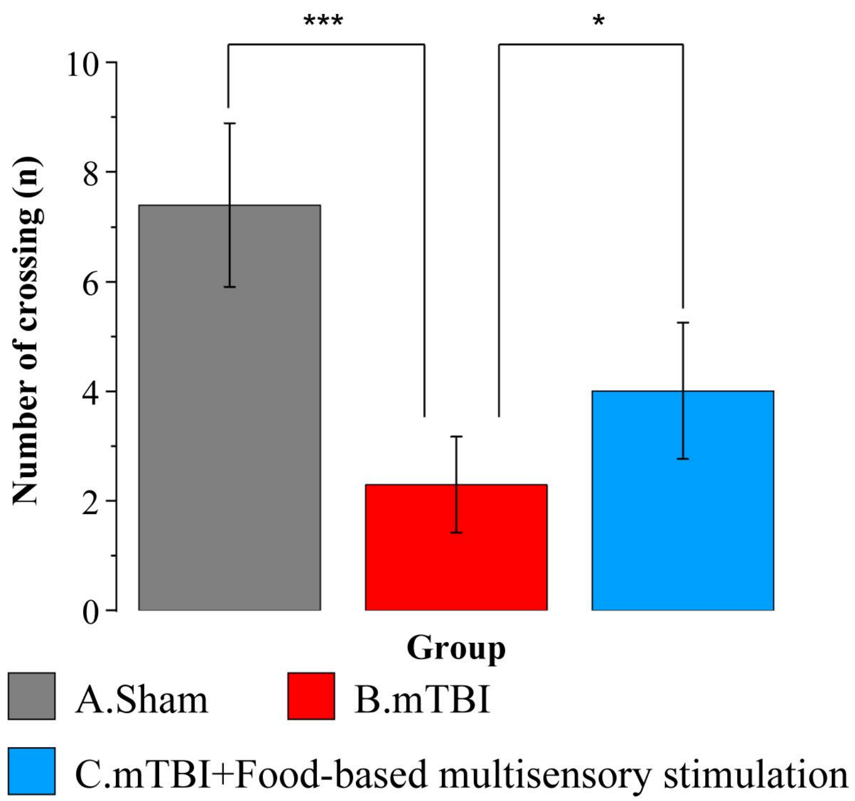
***
*
10
8
6
4
2
0
Number of crossing (n)
Group
A.Sham
B.mTBI
C.mTBI+Food-based multisensory stimulation

Fig 2 D

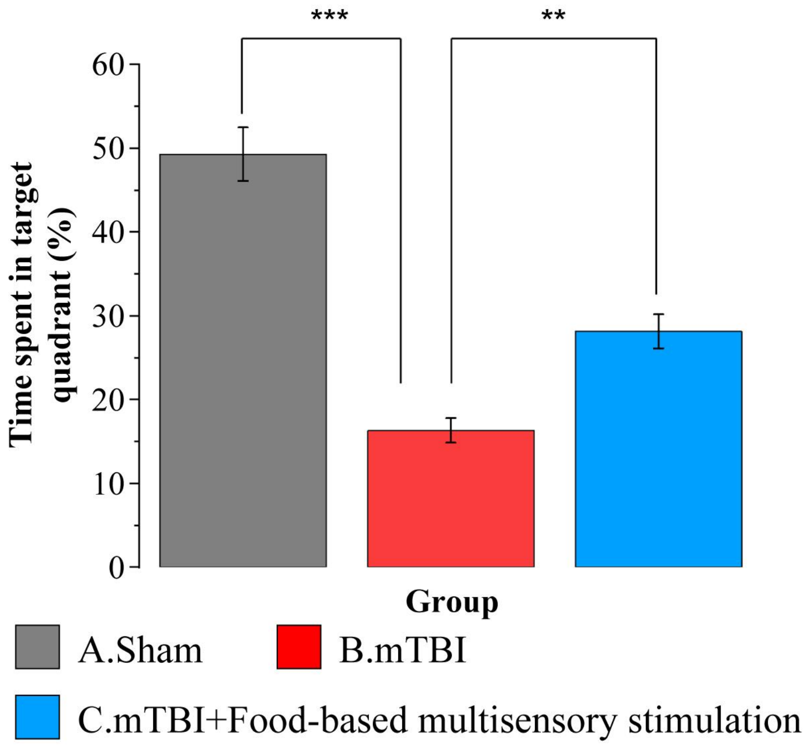
***
**
Time spent in target quadrant (%)
60
50
40
30
20
10
0
Group
A.Sham
B.mTBI
C.mTBI+Food-based multisensory stimulation

Fig 2 E

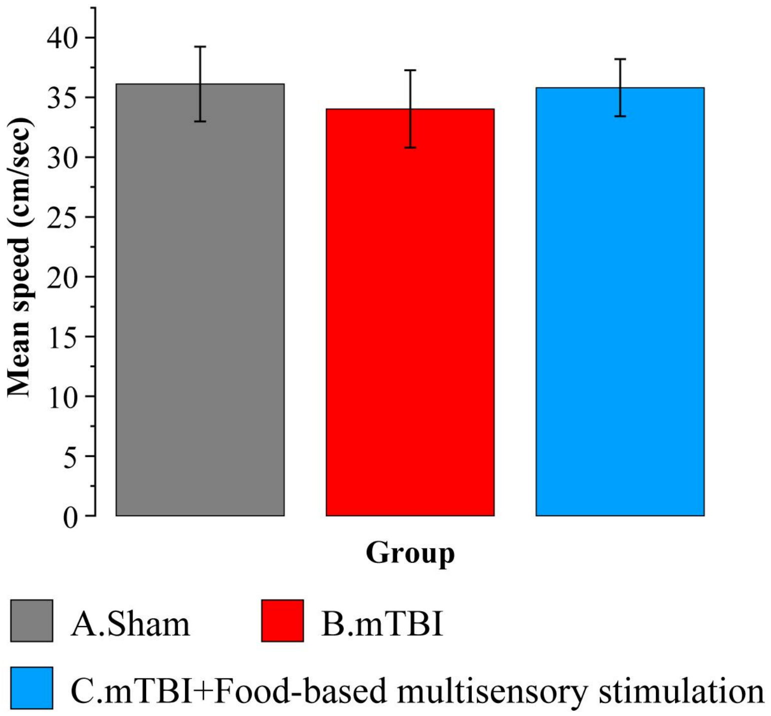

Mean speed (cm/sec)
40
35
30
25
20
15
10
5
0
Group
A.Sham
B.mTBI
C.mTBI+Food-based multisensory stimulation

Fig 2 F

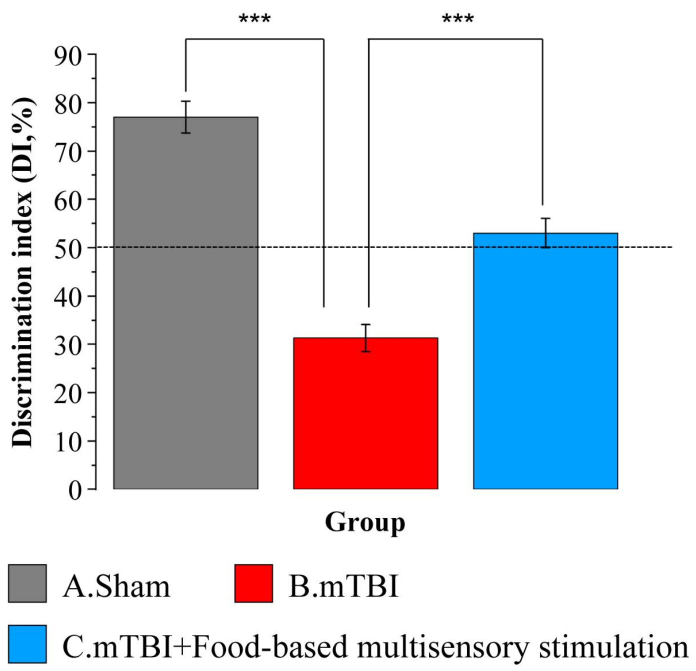
***
***
90
80
70
60
50
40
30
20
10
0
Discrimination index (DI,%)
Group
A.Sham
B.mTBI
C.mTBI+Food-based multisensory stimulation

Fig 2 G

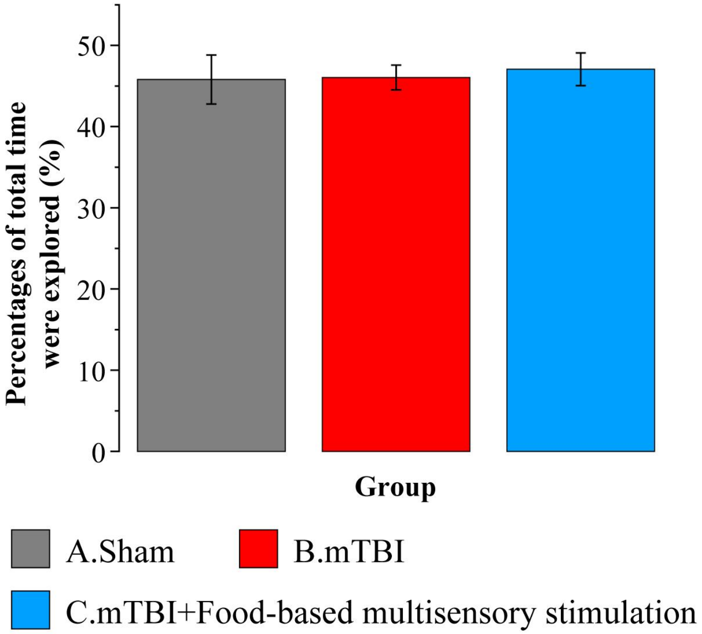

Percentages of total time were explored (%)
50
40
30
20
10
0
Group
A.Sham
B.mTBI
C.mTBI+Food-based multisensory stimulation

Fig 2 H

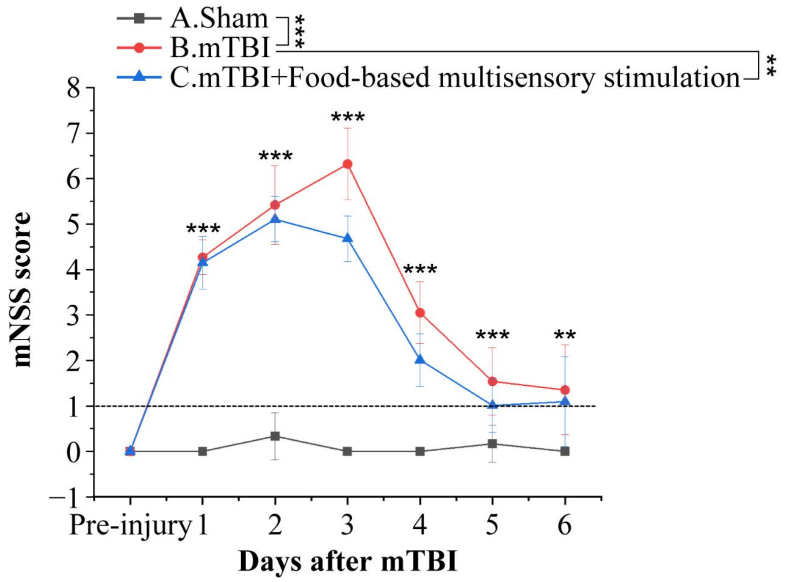

A.Sham
B.mTBI
C.mTBI+Food-based multisensory stimulation
***
**
mNSS score
Days after mTBI
Pre-injury
1
2
3
4
5
6
8
7
6
5
4
3
2
1
0
−1
***
***
***
***
***
**

Fig 2 I

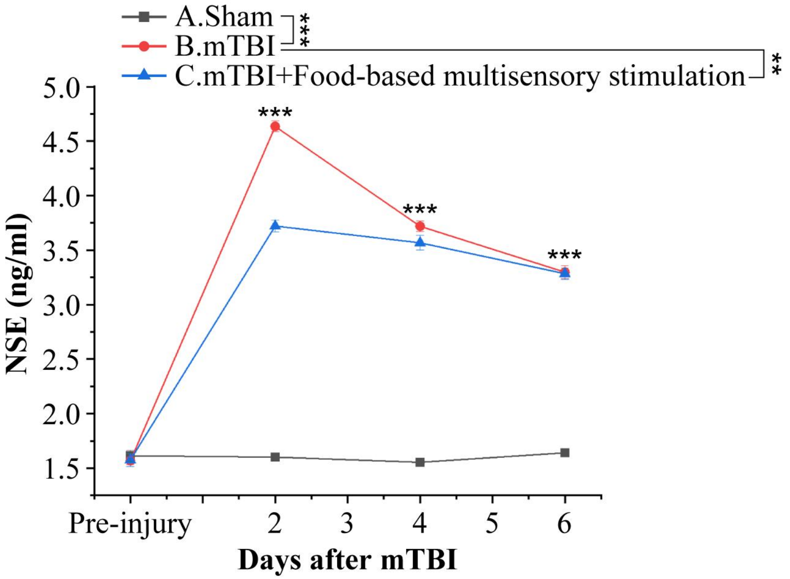
A.Sham
B.mTBI
C.mTBI+Food-based multisensory stimulation
***
**
5.0
4.5
4.0
3.5
3.0
2.5
2.0
1.5
NSE (ng/ml)
***
***
***
Pre-injury
2
3
4
5
6
Days after mTBI

Fig 2 J

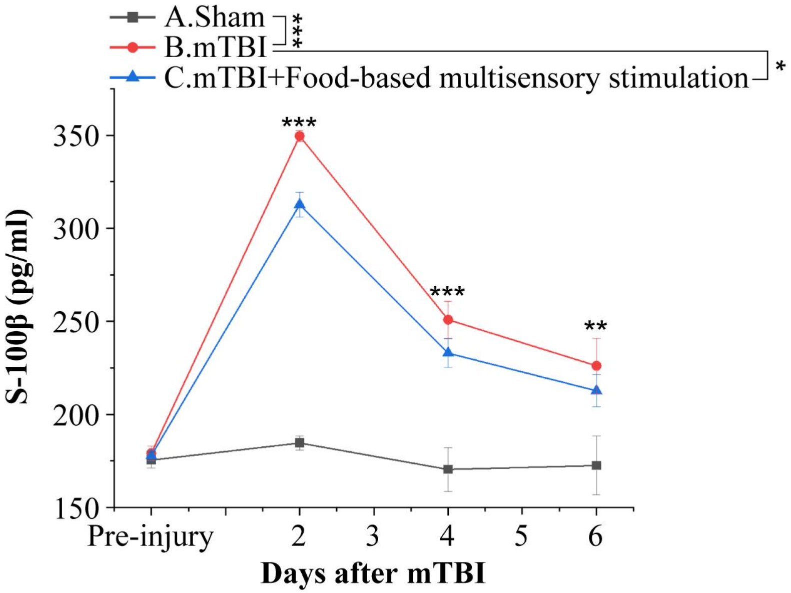

A.Sham
B.mTBI
C.mTBI+Food-based multisensory stimulation
***
*
***
***
**
350
300
250
200
150
S-100β (pg/ml)
Pre-injury
2
3
4
5
6
Days after mTBI

Fig 2 K

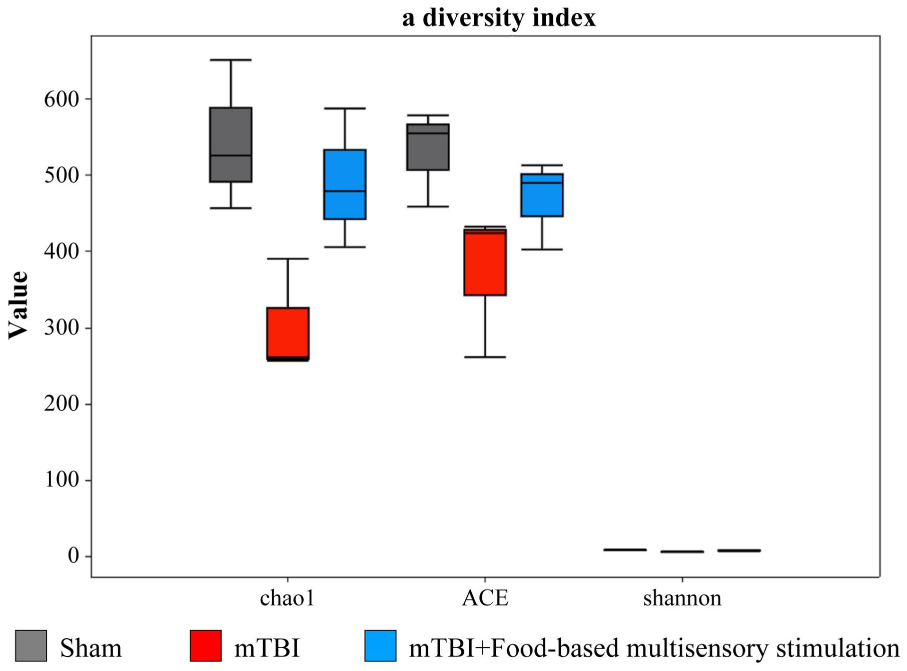
a diversity index
Value
600
500
400
300
200
100
0
chao1
ACE
shannon
Sham
mTBI
mTBI+Food-based multisensory stimulation

Fig 3 A

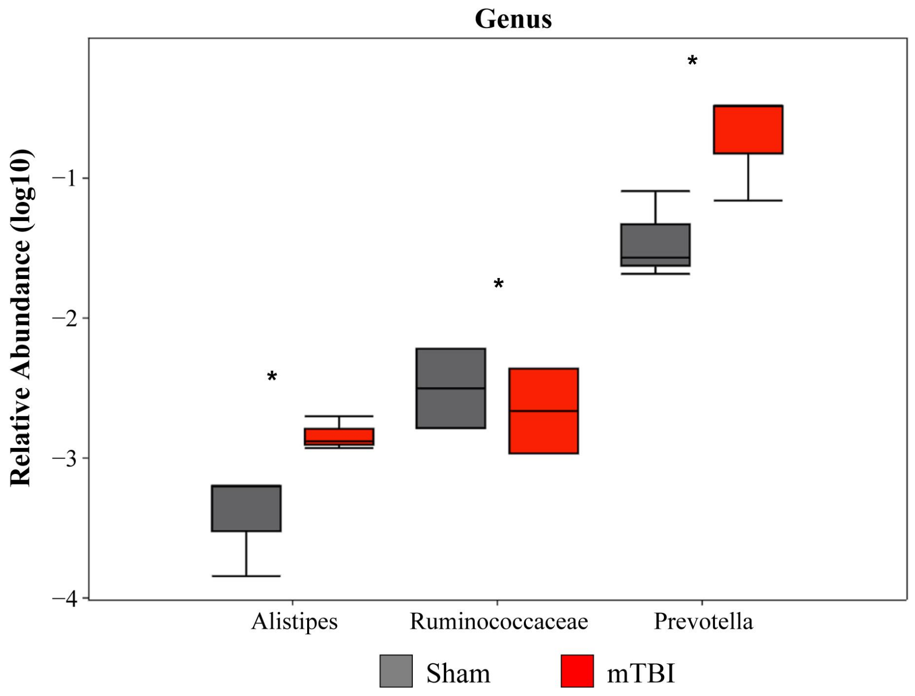

Genus
Relative Abundance (log10)
−1
−2
−3
−4
*
*
*
Alistipes
Ruminococcaceae
Prevotella
Sham
mTBI

Fig 3 B

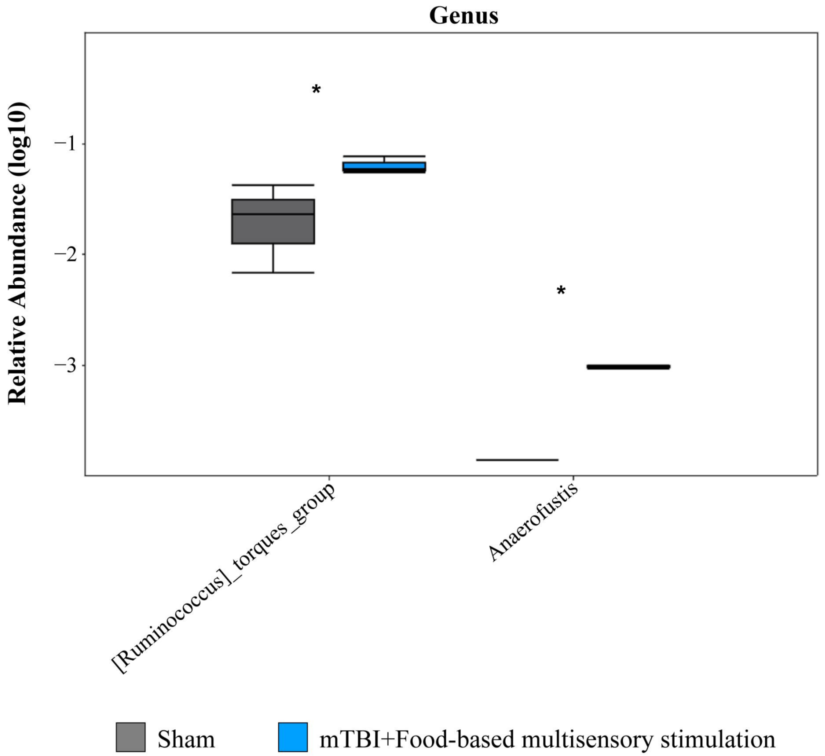
Genus
Relative Abundance (log10)
−1
−2
−3
*
*
[Ruminococcus]_torques_group
Anaerofustis
Sham
mTBI+Food-based multisensory stimulation

Fig 3 C

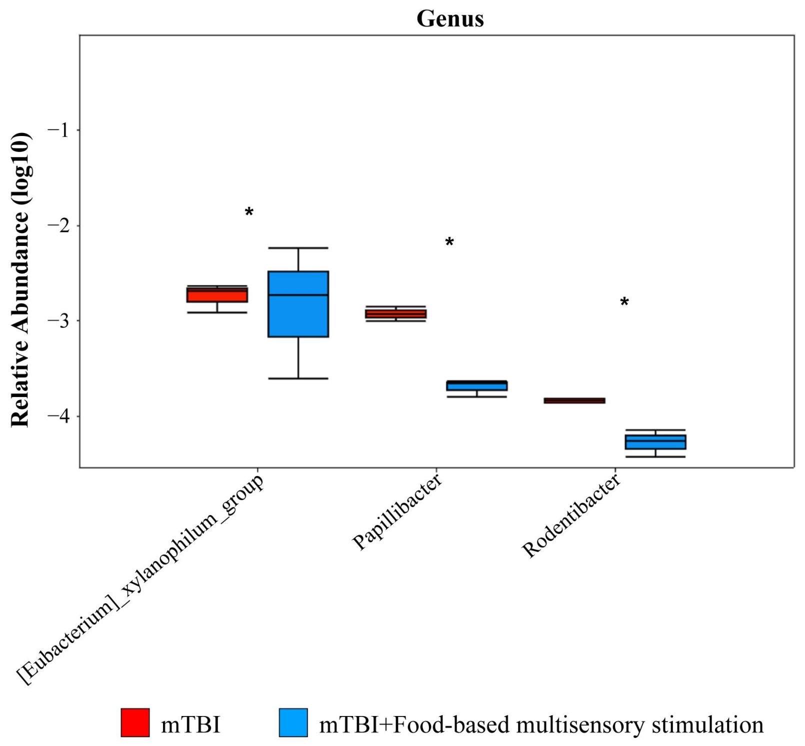

Genus
Relative Abundance (log10)
−1
−2
−3
−4
*
*
*
[Eubacterium]_xylanophilum_group
Papillibacter
Rodentibacter
mTBI
mTBI+Food-based multisensory stimulation

Fig 3 D

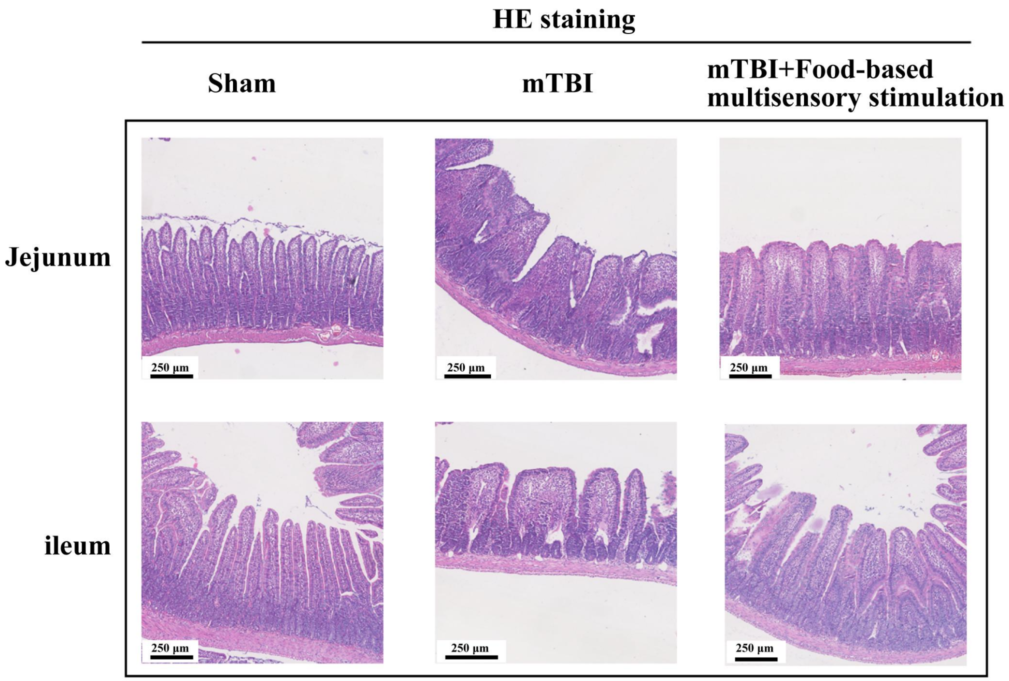
HE staining
Sham
mTBI
mTBI+Food-based multisensory stimulation
Jejunum
ileum
250 μm
250 μm
250 μm
250 μm
250 μm
250 μm

Fig 4 A

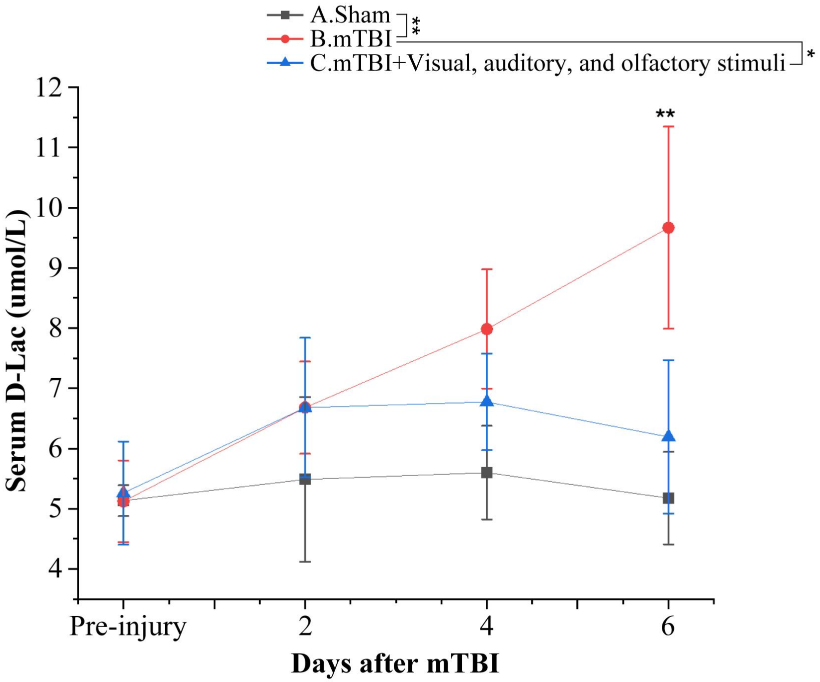
A.Sham
B.mTBI
C.mTBI+Visual, auditory, and olfactory stimuli
**
*
**
Serum D-Lac (umol/L)
12
11
10
9
8
7
6
5
4
Pre-injury
2
4
6
Days after mTBI

Fig 4 B

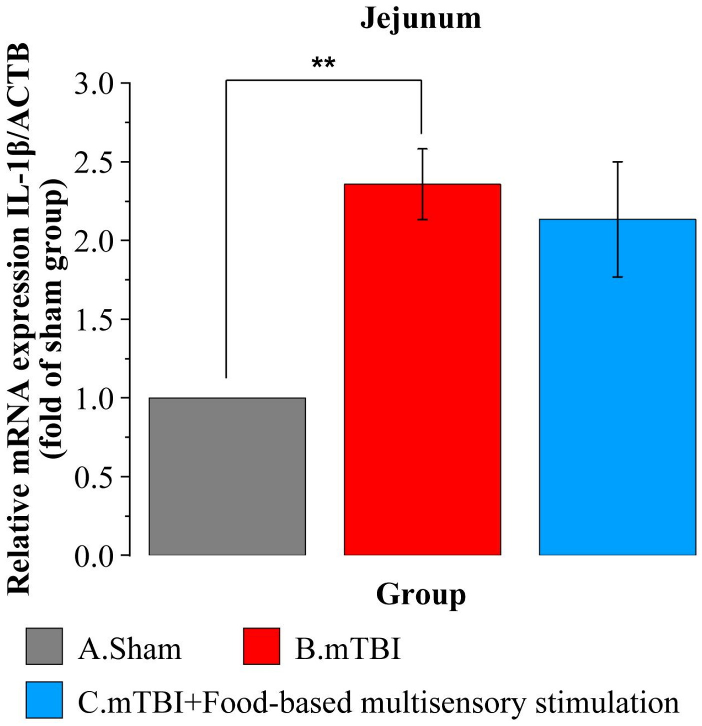
Jejunum
**
Relative mRNA expression IL-1β/ACTB
(fold of sham group)
3.0
2.5
2.0
1.5
1.0
0.5
0.0
Group
A.Sham
B.mTBI
C.mTBI+Food-based multisensory stimulation

Fig 4 C

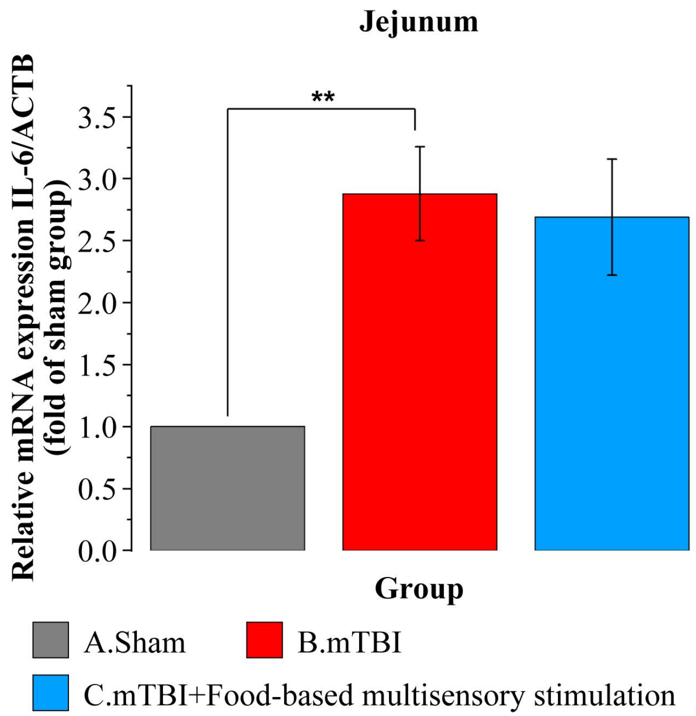
Jejunum
Relative mRNA expression IL-6/ACTB (fold of sham group)
**
3.5
3.0
2.5
2.0
1.5
1.0
0.5
0.0
Group
A.Sham
B.mTBI
C.mTBI+Food-based multisensory stimulation

Fig 4 D

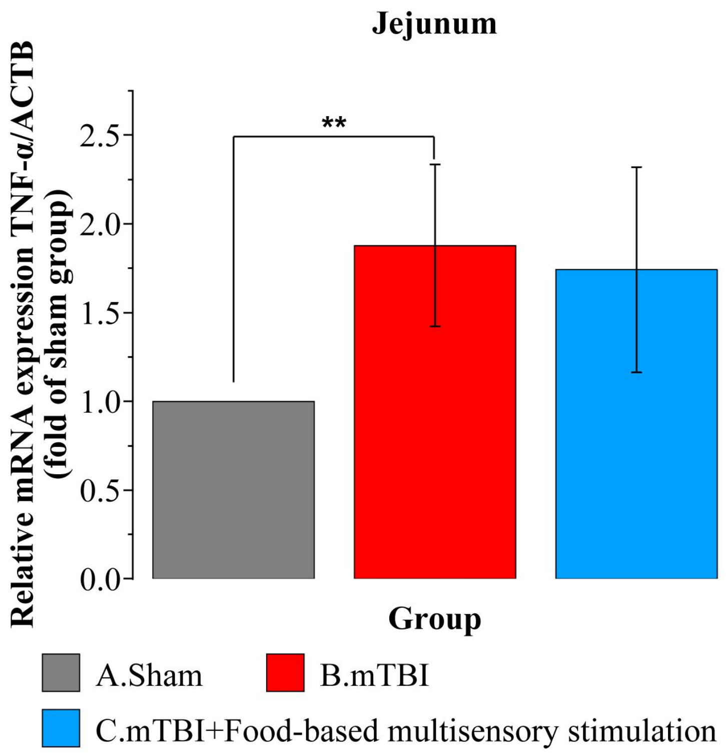
Jejunum
Relative mRNA expression TNF-α/ACTB (fold of sham group)
**
2.5
2.0
1.5
1.0
0.5
0.0
Group
A.Sham
B.mTBI
C.mTBI+Food-based multisensory stimulation

Fig 4 E

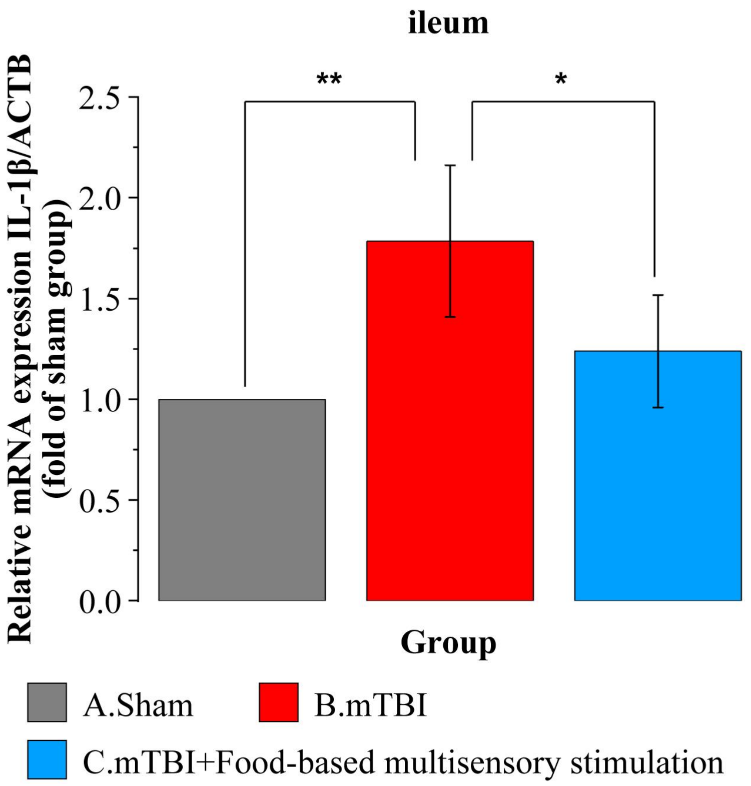

ileum
**
*
2.5
2.0
1.5
1.0
0.5
0.0
Relative mRNA expression IL-1β/ACTB
(fold of sham group)
Group
A.Sham
B.mTBI
C.mTBI+Food-based multisensory stimulation

Fig 4 F

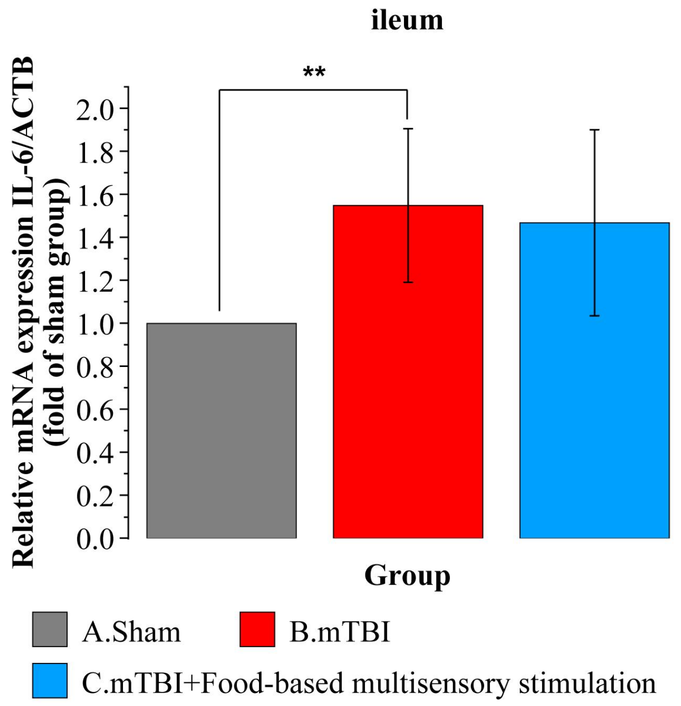
ileum
**
Relative mRNA expression IL-6/ACTB
(fold of sham group)
2.0
1.8
1.6
1.4
1.2
1.0
0.8
0.6
0.4
0.2
0.0
Group
A.Sham
B.mTBI
C.mTBI+Food-based multisensory stimulation

Fig 4 G

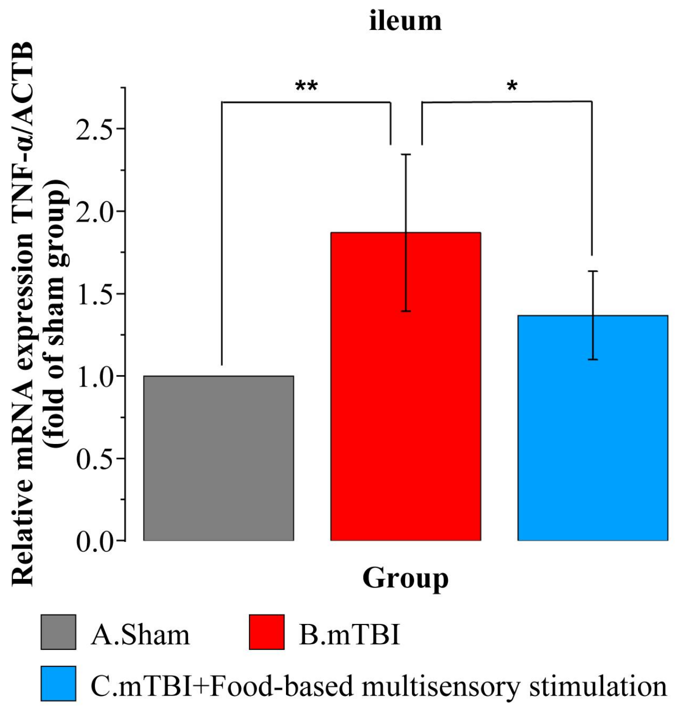
ileum
Relative mRNA expression TNF-α/ACTB
(fold of sham group)
2.5
2.0
1.5
1.0
0.5
0.0
**
*
Group
A.Sham
B.mTBI
C.mTBI+Food-based multisensory stimulation

Fig 4 H

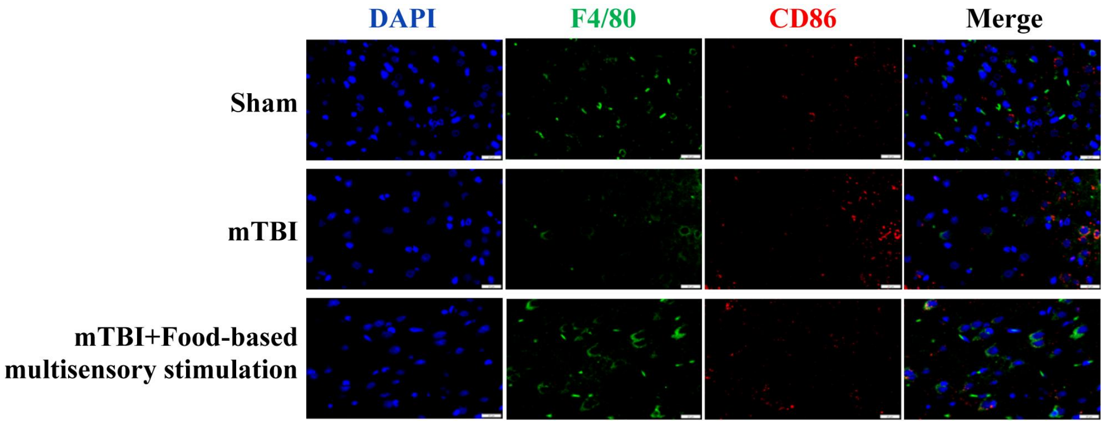

DAPI
F4/80
CD86
Merge
Sham
mTBI
mTBI+Food-based multisensory stimulation

Fig 5 A1

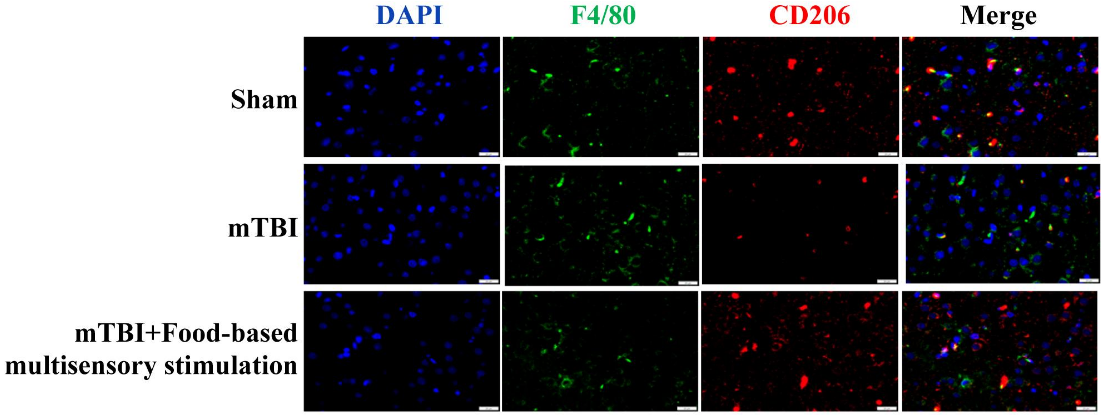
DAPI
F4/80
CD206
Merge
Sham
mTBI
mTBI+Food-based multisensory stimulation

Fig 5 A2

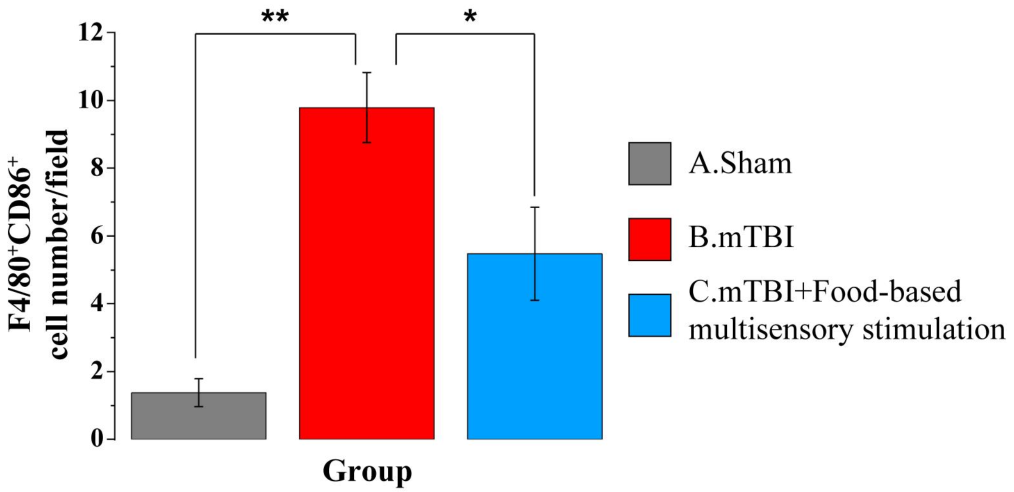

**
*
12
10
8
6
4
2
0
F4/80+CD86+
cell number/field
Group
A.Sham
B.mTBI
C.mTBI+Food-based
multisensory stimulation

Fig 5 B

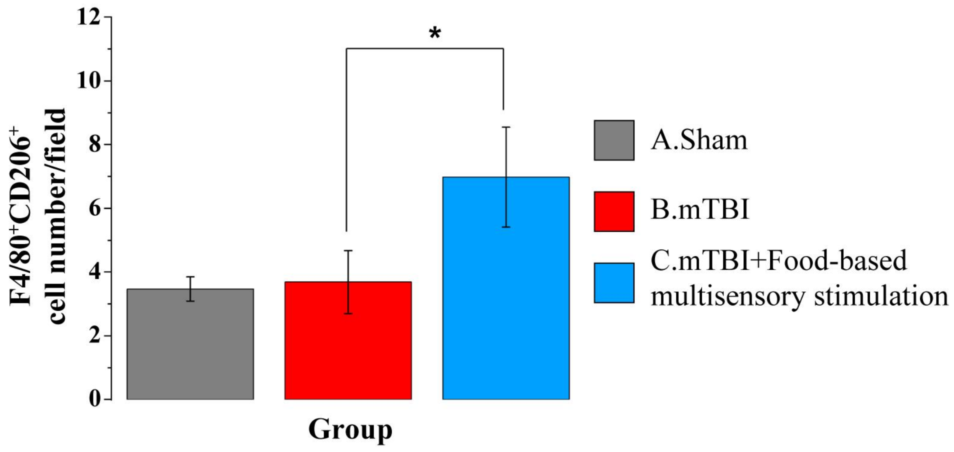

12
10
8
6
4
2
0
F4/80+CD206+ cell number/field
*
Group
A.Sham
B.mTBI
C.mTBI+Food-based multisensory stimulation

Fig 5 C

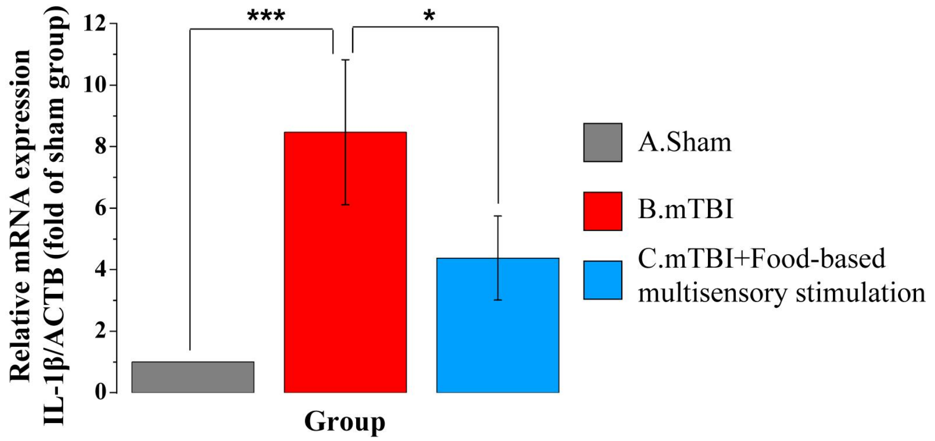
Relative mRNA expression
IL-1β/ACTB (fold of sham group)
12
10
8
6
4
2
0
***
*
Group
A.Sham
B.mTBI
C.mTBI+Food-based
multisensory stimulation

Fig 5 D

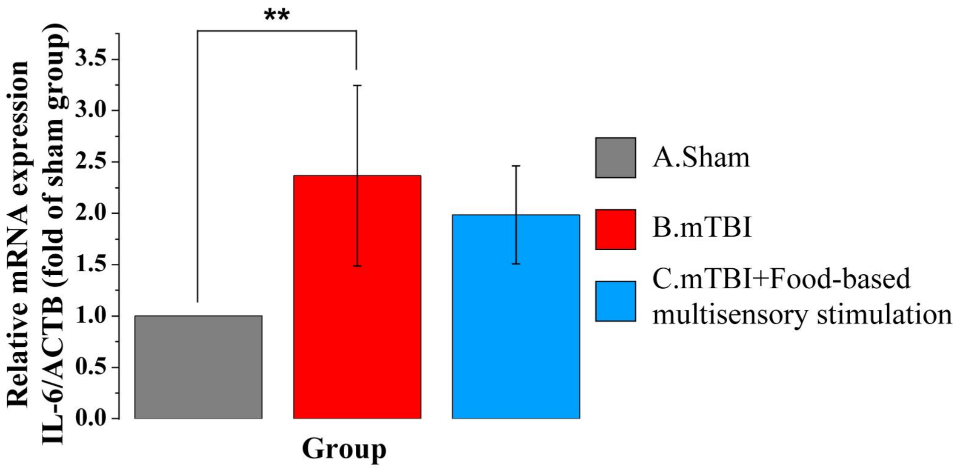

**
Relative mRNA expression
IL-6/ACTB (fold of sham group)
3.5
3.0
2.5
2.0
1.5
1.0
0.5
0.0
Group
A.Sham
B.mTBI
C.mTBI+Food-based multisensory stimulation

Fig 5 E

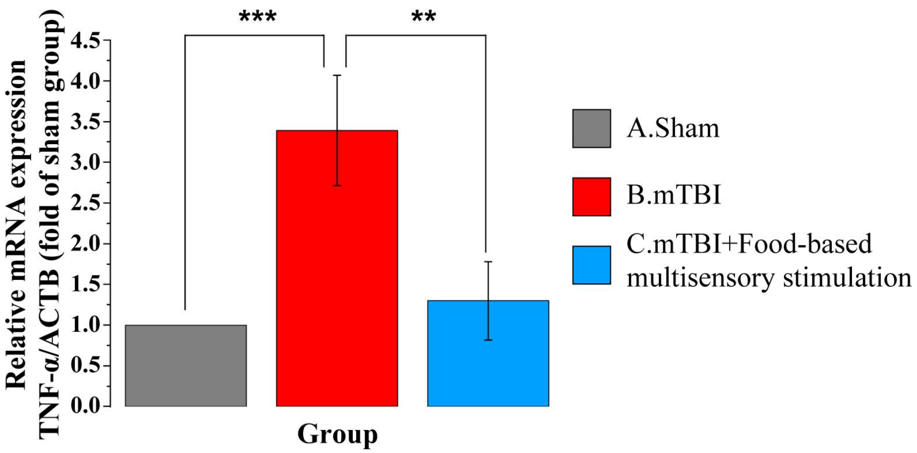
***
**
Relative mRNA expression
TNF-α/ACTB (fold of sham group)
4.5
4.0
3.5
3.0
2.5
2.0
1.5
1.0
0.5
0.0
Group
A.Sham
B.mTBI
C.mTBI+Food-based
multisensory stimulation

Fig 5 F

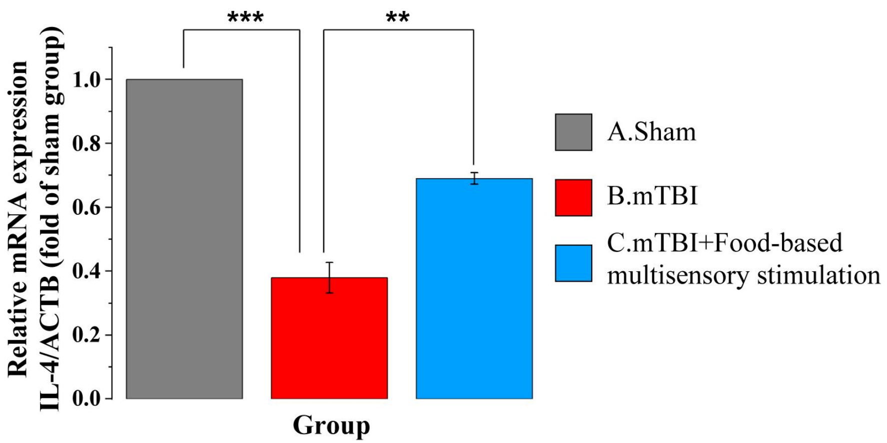
***
**
Relative mRNA expression
IL-4/ACTB (fold of sham group)
1.0
0.8
0.6
0.4
0.2
0.0
Group
A.Sham
B.mTBI
C.mTBI+Food-based
multisensory stimulation

Fig 5 G

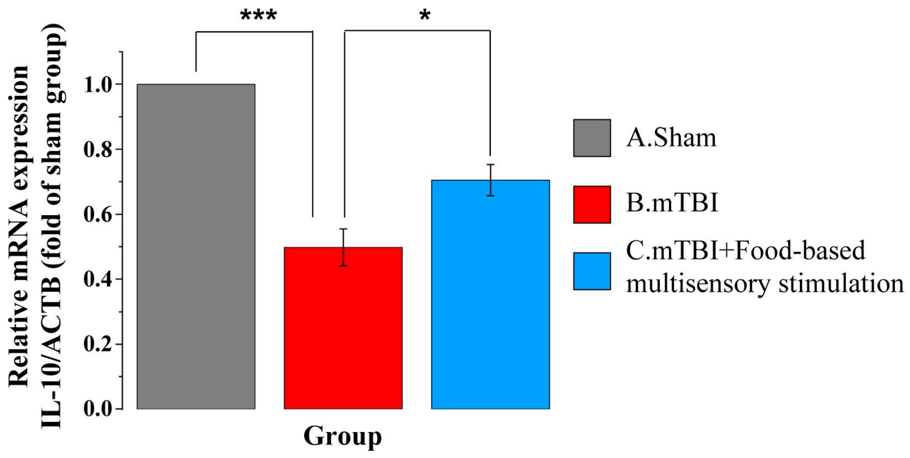
***
*
Relative mRNA expression
IL-10/ACTB (fold of sham group)
1.0
0.8
0.6
0.4
0.2
0.0
Group
A.Sham
B.mTBI
C.mTBI+Food-based
multisensory stimulation

Fig 5 H

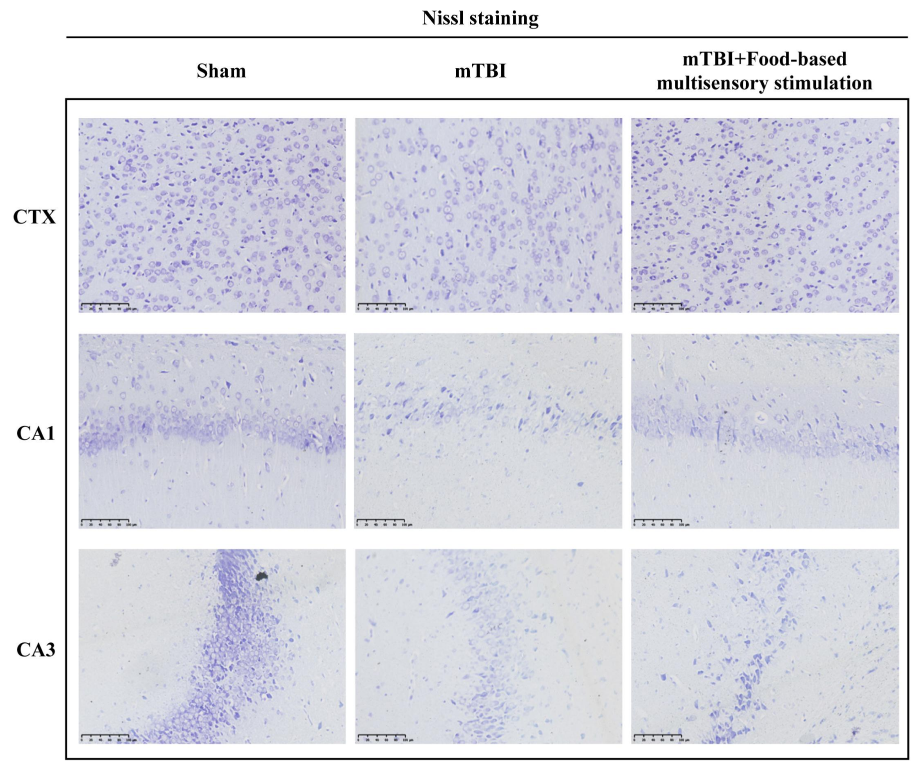
Nissl staining
Sham
mTBI
mTBI+Food-based multisensory stimulation
CTX
CA1
CA3

Fig 5 I

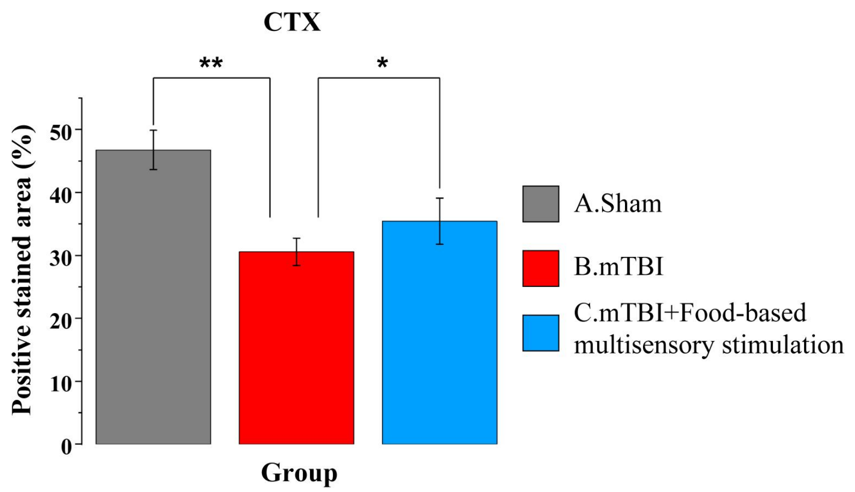
CTX
**
*
Positive stained area (%)
50
40
30
20
10
0
Group
A.Sham
B.mTBI
C.mTBI+Food-based
multisensory stimulation

Fig 5 J

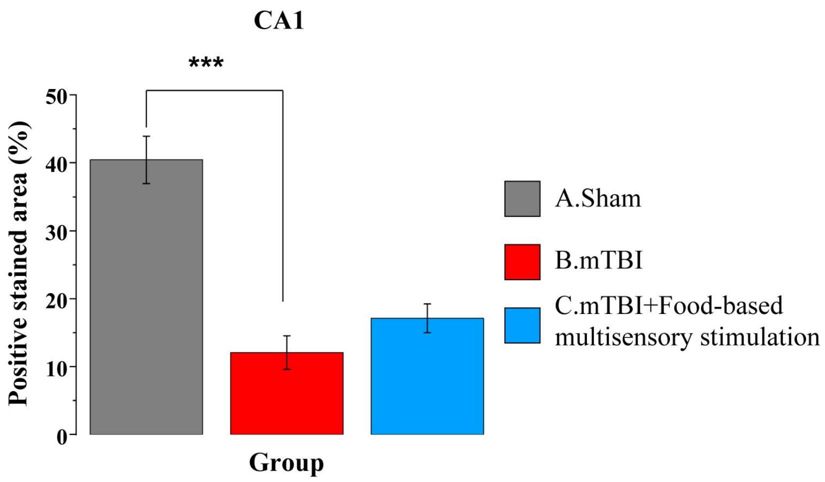
CA1
***
Positive stained area (%)
50
40
30
20
10
0
Group
A.Sham
B.mTBI
C.mTBI+Food-based multisensory stimulation

Fig 5 K

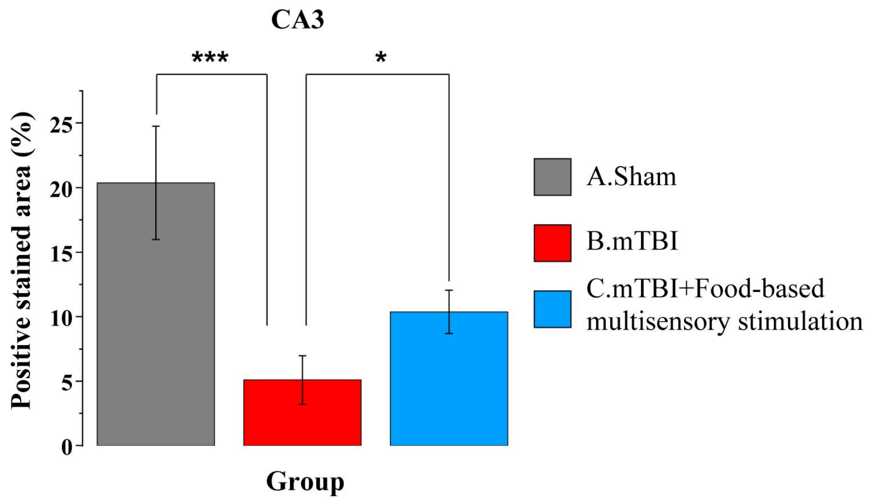

CA3
***
*
Positive stained area (%)
25
20
15
10
5
0
Group
A.Sham
B.mTBI
C.mTBI+Food-based multisensory stimulation

Fig 5 L

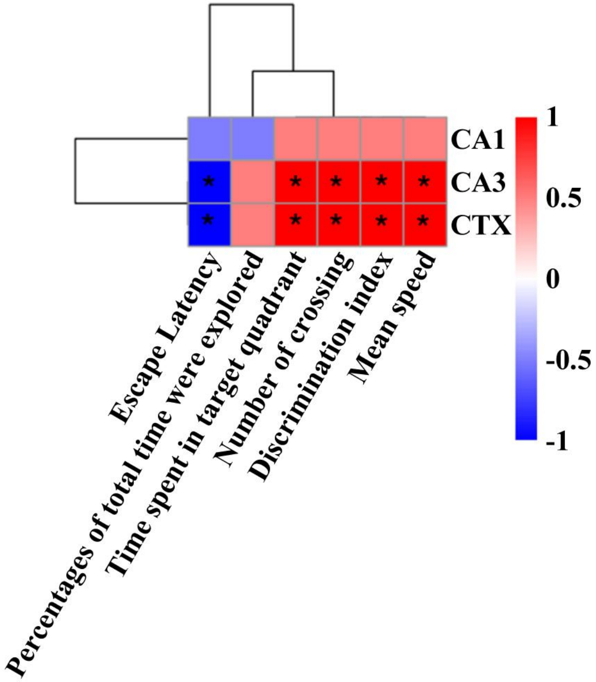

CA1
CA3
CTX
Escape Latency
Percentages of total time were explored
Time spent in target quadrant
Number of crossing
Discrimination index
Mean speed
1
0.5
0
-0.5
-1

Fig 5 M

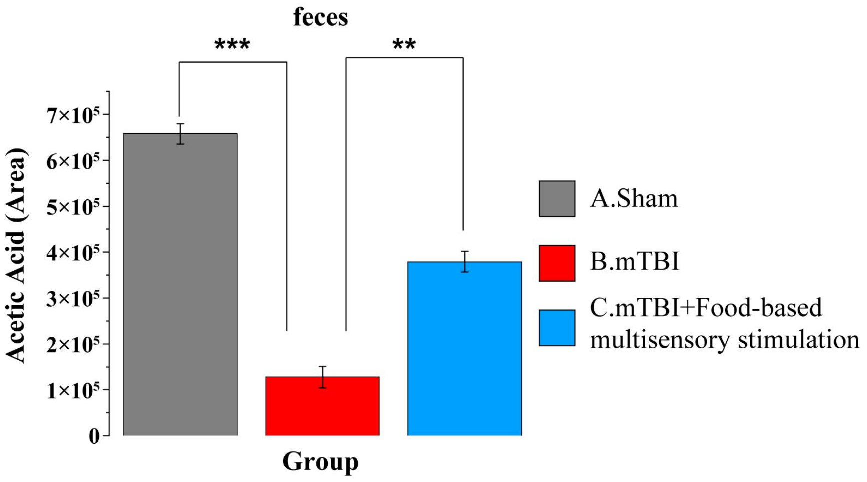
feces
***
**
Acetic Acid (Area)
7×10^5
6×10^5
5×10^5
4×10^5
3×10^5
2×10^5
1×10^5
0
Group
A.Sham
B.mTBI
C.mTBI+Food-based multisensory stimulation

Fig 6 A1

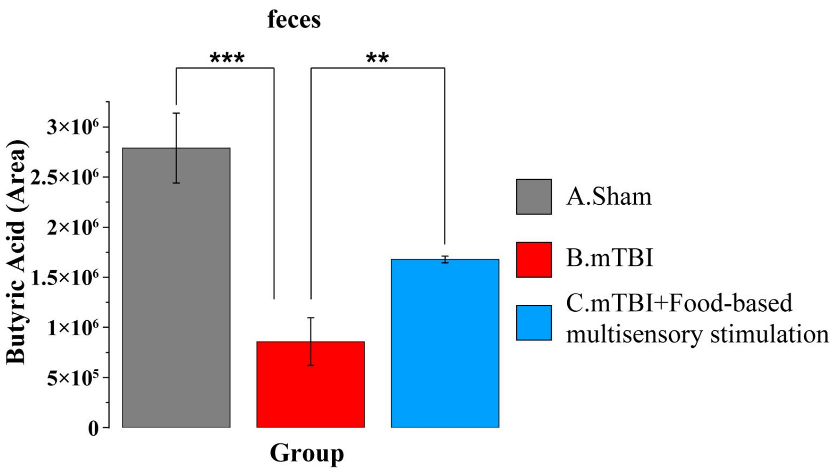
feces
***
**
Butyric Acid (Area)
3×10⁶
2.5×10⁶
2×10⁶
1.5×10⁶
1×10⁶
5×10⁵
0
Group
A.Sham
B.mTBI
C.mTBI+Food-based multisensory stimulation

Fig 6 A2

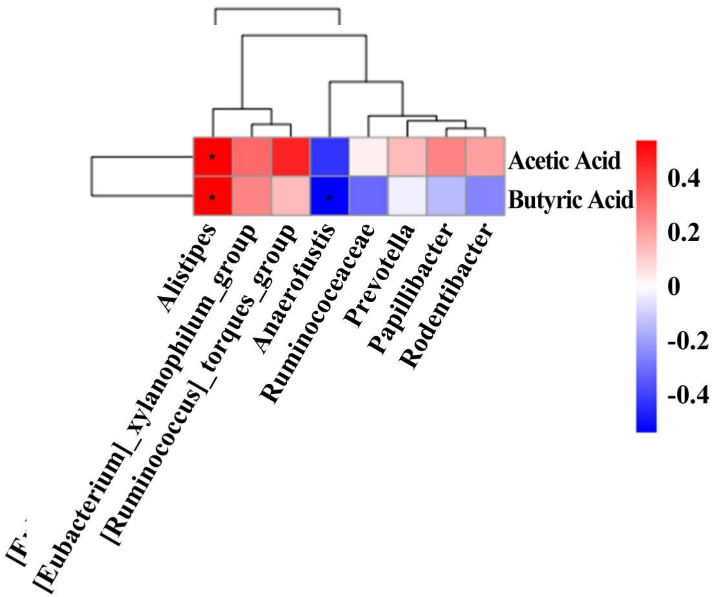

Acetic Acid
Butyric Acid
Alistipes
[Eubacterium]_xylanophilum_group
[Ruminococcus]_torques_group
Anaerofustis
Ruminococcaceae
Prevotella
Papillibacter
Rodentibacter
0.4
0.2
0
-0.2
-0.4

Fig 6 A3

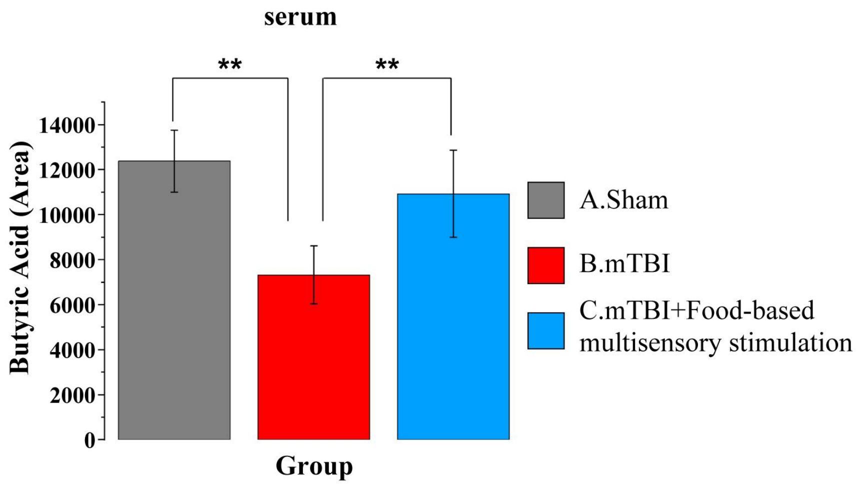
serum
**
**
14000
12000
10000
8000
6000
4000
2000
0
Butyric Acid (Area)
Group
A.Sham
B.mTBI
C.mTBI+Food-based
multisensory stimulation

Fig 6 A4

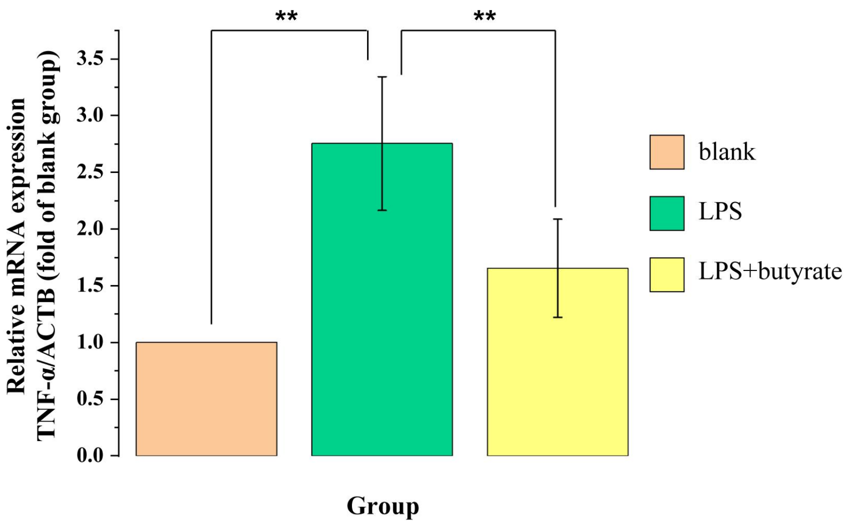
**
**
3.5
3.0
2.5
2.0
1.5
1.0
0.5
0.0
Relative mRNA expression
TNF-α/ACTB (fold of blank group)
Group
blank
LPS
LPS+butyrate

Fig 6 B1

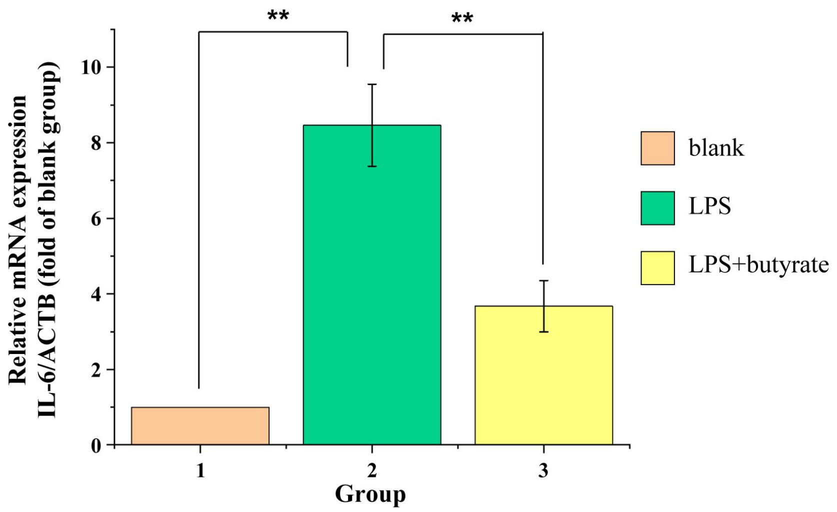
Relative mRNA expression
IL-6/ACTB (fold of blank group)
**
**
0
2
4
6
8
10
1
2
3
Group
blank
LPS
LPS+butyrate

Fig 6 B2

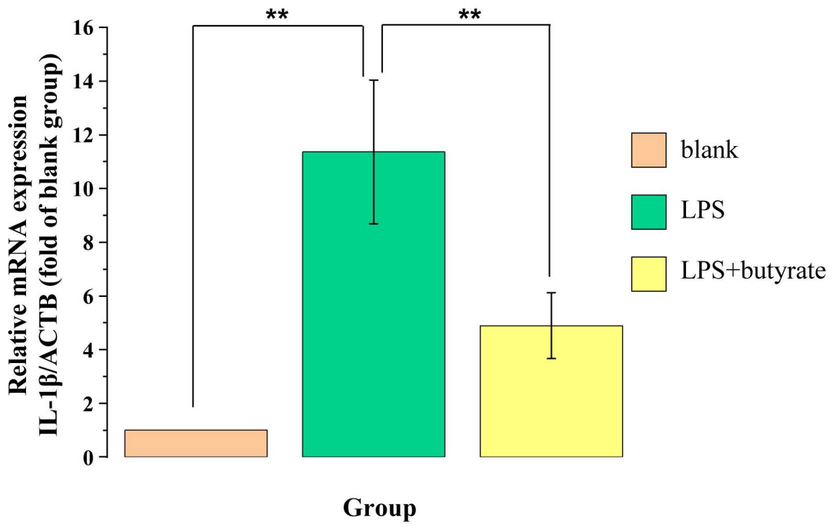

Relative mRNA expression
IL-1β/ACTB (fold of blank group)
0
2
4
6
8
10
12
14
16
**
**
Group
blank
LPS
LPS+butyrate

Fig 6 B3

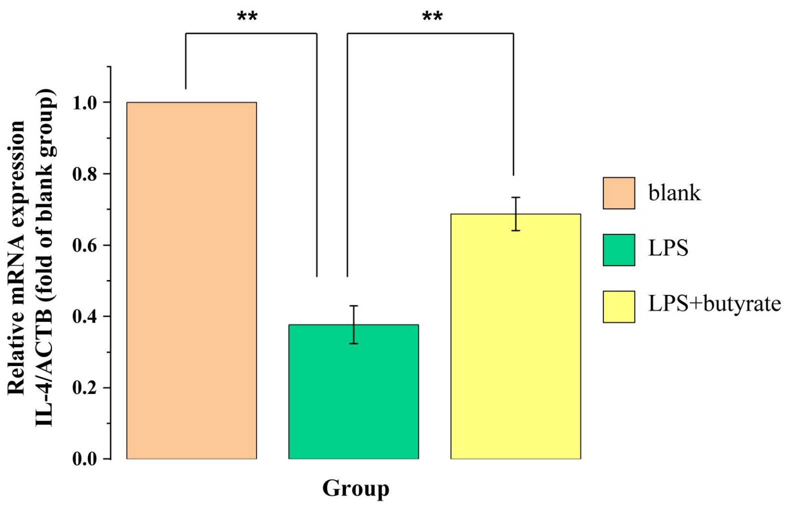
**
**
1.0
0.8
0.6
0.4
0.2
0.0
Relative mRNA expression
IL-4/ACTB (fold of blank group)
Group
blank
LPS
LPS+butyrate

Fig 6 B4

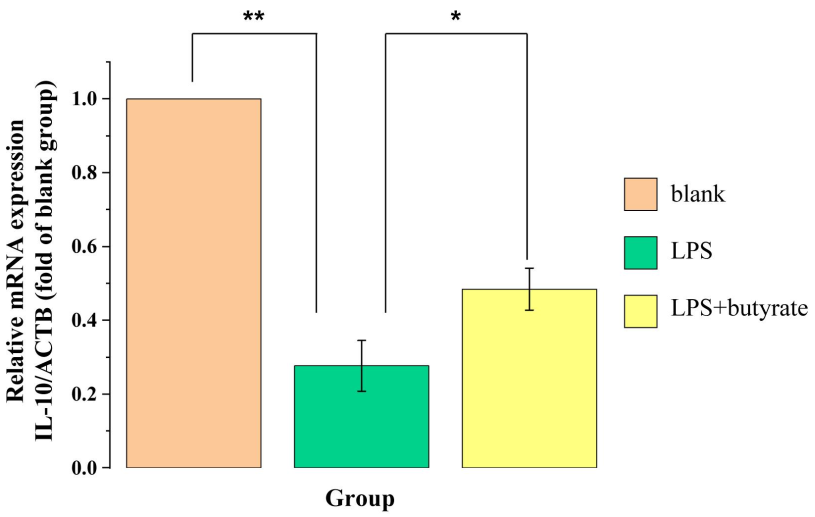

**
*
Relative mRNA expression
IL-10/ACTB (fold of blank group)
0.0
0.2
0.4
0.6
0.8
1.0
Group
blank
LPS
LPS+butyrate

Fig 6 B5
