# Supplementary material for: Food-based multisensory stimulation ameliorates cognitive impairment after mild traumatic brain injury in male rats by modulating intestinal and brain inflammation
Source: PLoS One. 2026 Feb 27;21(2):e0343814. doi: 10.1371/journal.pone.0343814 (PMC12948070; doi:10.1371/journal.pone.0343814)
Supplement: Attachment — Submitted filename: Comments PONE-D-25-51866.docx [file pone.0343814.s004.docx]

This manuscript presents a promising approach to treating cognitive impairment after mild traumatic brain injury using food-based multisensory stimulation. The in vivo work is generally well-executed with comprehensive behavioral, microbiome, and histological analyses that convincingly demonstrate therapeutic benefits. However, the study suffers from several critical weaknesses.

1. The planning of the cognitive experiments (Morris water and nort) in the early post-TBI days may be confounded by sensorimotor impairments and neurological severity, making it difficult to accurately assess cognitive function. The authors should clarify how they accounted for this potential confound.
2. The introduction should be revised to clearly and concisely address the gut–brain connection in mTBI, the associated cognitive impairments, and the rationale for exploring food-based interventions in TBI (PMID: 36481824, 39751900).
3. What was the exact timeline for all the experiments in sequence? A study design figure depicting the time should be made.
4. For the estimation of NSE and S-100β, the same animals were used for different timepoints or different animals, and this should be clearly stated in the methods section.
5. Interventions commenced immediately post-injury and continued for seven days, operating around the clock. But there is no rationale for why 7 days were chosen. No citation of previous multisensory stimulation studies using this duration. No pilot data mentioned. No dose-finding or optimization experiments. The choice appears arbitrary.
6. Also 7 days captures only the acute phase, Gut microbiome remodeling typically takes 2-4 weeks to stabilize
7. The idea of this work revolves around the food based multisensory stimulation. So, was the amount of food consumed by each animal noted throughout the study plan, and what about the weight of the animals data?
8. In the relative mRNA expression results, what is ACTB?
9. The Nissl-positive area has significantly improved in the food-based multisensory group in the CTX and CA3 regions, but not in the CA1 region. What could be the reason?
10. Was an established protocol followed for conducting the behavioral experiments? The method used for calculating the Discrimination Index appears to be adapted from previously published protocols, and therefore, relevant literature should be referenced (e.g., PMID: 38423243).
11. Please clarify how the food items (pet biscuits, chicken jerky) were selected and provide their nutritional composition. Also specify whether the scents were standardized, the concentrations used, and the volume/intensity of the chewing sounds. Indicate whether rats had physical access to the food or only sensory exposure. A brief, detailed protocol describing all stimulus parameters (duration, concentration, intensity) and a simple methods figure of the setup would improve clarity.
12. While the in vitro data (Fig. 4O) shows anti-inflammatory effects of butyrate, it does not establish that butyrate mediates the in vivo cognitive benefits of sensory stimulation; the authors should either include a butyrate-only or butyrate-blockade group, or clearly acknowledge this limitation in the discussion.
13. With numerous outcome measures across multiple timepoints and tissue types, correction for multiple comparisons should be applied or justified if not applied
14. A sensory stimulation + sham injury group would help determine if the intervention affects healthy animals or is specific to mTBI pathology. Either add this control group or explicitly discuss this limitation and its implications for interpreting the specificity of the intervention.
15. The correlation between specific taxa and SCFAs is interesting, but doesn't prove causality. Consider softening language.
16. The differential effects across brain regions (cortex and CA3 protected, CA1 not protected) are intriguing but underexplored. Discuss potential mechanisms for regional specificity. Could it relate to differential expression of SCFA receptors (GPR41, GPR43) or regional vulnerability to the injury model?
17. Abstract: "may be associated" and "possibly promoting" are appropriately cautious but could be stronger given your data. Consider removing some hedging language where data directly support conclusions
18. Methods section numbering is inconsistent (2.4 appears twice)
19. What was the microglial cell source, primary cells? (from rats? mice?) or Cell line? (BV2? N9? …..)
20. Consider discussing the acute (7-day) vs. chronic nature of the intervention and whether effects would persist after cessation
21. In Figure 3, HE staining images need scale bars. Quantification of villus height, crypt depth, or villi to crypt ratio and goblet cell density would strengthen conclusions.
22. Figure 4 is dense with information, consider splitting it into two figures. The correlation heatmap needs clearer labeling
23. Could isoflurane exposure affect gut microbiota? Did sham animals receive equivalent anesthesia duration?
24. How were fecal samples collected to avoid environmental contamination? Were samples from individual pellets or the cage floor?
25. Only male rats were used. Justify this choice and discuss how results might differ in females, given known sex differences in TBI outcomes and microbiome composition.
26. Were animals group housed or single housed? This significantly affects the microbiome.
27. The sample size for each experiment and each group should be indicated in the figure legends to ensure transparency, rigor, and reproducibility of the data.
28. Consider revisiting the manuscript to avoid typos and grammatical errors.
