# Supplementary material for: Food-based multisensory stimulation ameliorates cognitive impairment after mild traumatic brain injury in male rats by modulating intestinal and brain inflammation
Source: PLoS One. 2026 Feb 27;21(2):e0343814. doi: 10.1371/journal.pone.0343814 (PMC12948070; doi:10.1371/journal.pone.0343814)
Supplement: Attachment — Submitted filename: Response to reviewers.docx [file pone.0343814.s005.docx]

**Response to reviewers**

Academic Editor: 
1) Ideally, all animal research should conform to ARRIVE guidelines (https://journals.plos.org/plosbiology/article?id=10.1371/journal.pbio.3000410) Please incorporate these guidelines in your manuscript and I would strongly suggest attaching a completed guideline checklist as supplemental material.

**Responses**: Thank you for the suggestion. We have now incorporated the ARRIVE 2.0 guidelines into the manuscript and submitted a completed ARRIVE checklist as supplementary material

2) How did you control for bias in your experimentations?  Was blinding used?  If so, please indicate when and where.  If not, please also detail this.

**Responses:** We thank the reviewer for this important comment. In the revised manuscript, we have now provided a more detailed description of the measures taken to reduce bias. Group allocation was performed by an investigator who did not take part in the subsequent behavioural testing or data analysis, and the investigators who conducted the behavioural assessments and analysed the data were blinded to group assignment. This information has been added to the Methods section of the manuscript.

3) You seem to have exclusively used male animals.  Why is that?  At a minimum, the title should reflect this and you should state this in your study limitations.

**Responses:** We thank the reviewer for raising this important point. In the present study, we used only male rats for several reasons. First, we aimed to minimise hormonal and behavioural variability related to the oestrous cycle, which may markedly influence cognitive performance in behavioural tests as well as stress-related physiological responses. Second, our primary objective in this initial set of experiments was to establish the feasibility and efficacy of a food‑based sensory stimulation intervention in a mild traumatic brain injury model in a relatively homogeneous cohort of animals. In line with the reviewer’s suggestion, we have (i) revised the title to indicate that the experiments were conducted in male rats and (ii) added a statement in the “Study limitations” section of the Discussion, noting that the inclusion of only males may limit the generalisability of our findings to females and that future studies should include both male and female animals.

4) Your discussion has no study limitations.  Traditionally, this would be the paragraph just before the conclusion where you outline all of the limitations to your study.  Please include this.

**Responses:** We thank the editor for this helpful comment. In the revised manuscript, we have now added a separate paragraph on the limitations of our study in the Discussion section, placed immediately before the Conclusion.

5) How did you control for confounders?  Please outline this in the methods.

**Responses:** We appreciate this important comment. In the revised manuscript, we have now explicitly described the measures taken to minimise potential confounders. Briefly, all animals were housed under identical environmental conditions, and group allocation was randomised and performed by an investigator who was not involved in behavioural testing or data analysis. The investigators conducting the behavioural assessments and statistical analyses were blinded to group allocation. All animals underwent the same surgical procedures and perioperative care, sham-operated rats were handled identically except for the weight-drop injury, and behavioural and sampling time points were standardised across groups.

6) In the results section, for comparisons, please list the F statistic for the group, and please list any p value for any comparison that you make.

**Responses**: Thank you for the reviewer’s suggestion. Due to space limitations in the Results section, listing the F statistics and p values for every single comparison in the main text would make the section overly long and reduce readability. Therefore, we report these statistics in the main text only for the key/primary comparisons. Meanwhile, we have compiled and uploaded the complete set of F statistics and p values (original statistical outputs) for all comparisons as Supplementary Data for the reviewer’s and readers’ reference and verification.
7) In your figures, you compare the sham group to the TBI-control group, and you compare the TBI-control group to the TBI+multisensory stim group.  Please also show the comparisons between the sham group and the TBI+multisensory stim group.

**Responses:** We appreciate the reviewer’s careful reading of our figures and the suggestion to include additional comparisons. In the present study, our primary objective was to (1) confirm the effect of mTBI by comparing the sham and TBI-control groups, and (2) evaluate whether multisensory stimulation could ameliorate the deficits induced by mTBI by comparing the TBI-control and TBI+multisensory stimulation groups. Therefore, our a priori hypothesis and statistical design focused on these two planned comparisons rather than on differences between the sham and TBI+multisensory stimulation groups.

We agree that the sham vs. TBI+multisensory stimulation comparison may be of descriptive interest; however, it was not part of our original hypothesis, and adding multiple post‑hoc comparisons beyond the predefined analytical plan could increase the risk of type I error and dilute the focus of the study. For this reason, we have not emphasized this comparison in the main figures.

8) At several points, you use the verb "expression" or the phrase "expression levels" in relation to mRNA.  This is confusing.  Protein is generally described as having expression levels.  For mRNA, please remove the term "expression."

**Responses**: We thank the reviewer for this helpful clarification. In the revised manuscript, we have replaced phrases such as “mRNA expression” and “mRNA expression levels” with more precise terminology.

==============================

Journal requirements:

When submitting your revision, we need you to address these additional requirements.

1. Please ensure that your manuscript meets PLOS ONE's style requirements, including those for file naming. The PLOS ONE style templates can be found at

https://journals.plos.org/plosone/s/file?id=wjVg/PLOSOne_formatting_sample_main_body.pdf and

https://journals.plos.org/plosone/s/file?id=ba62/PLOSOne_formatting_sample_title_authors_affiliations.pdf

**Responses**: Thank you for the reminder. We have revised the manuscript and file naming to conform to PLOS ONE style, following the official formatting templates.

2. To comply with PLOS One submissions requirements, in your Methods section, please provide additional information regarding the experiments involving animals and ensure you have included details on (1) methods of sacrifice, (2) methods of anesthesia and/or analgesia, and (3) efforts to alleviate suffering.

**Responses:** We thank the editor for this comment. The requested information on methods of sacrifice, anesthesia/analgesia, and efforts to alleviate suffering has now been added to the Methods section in the revised manuscript.

3. Thank you for stating the following financial disclosure:

 [This research was funded by the Regional joint key projects of NSFC (No. U22A20334), and Sichuan Province Science and Technology Support Program (2024NSFSC0592).].

Please state what role the funders took in the study.  If the funders had no role, please state: "The funders had no role in study design, data collection and analysis, decision to publish, or preparation of the manuscript."

If this statement is not correct you must amend it as needed.

Please include this amended Role of Funder statement in your cover letter; we will change the online submission form on your behalf.

**Responses**: Thank you for your guidance regarding the Role of the Funder statement. We have now added the following statement to the manuscript:
“This research was funded by the Regional joint key projects of NSFC (No. U22A20334) and the Sichuan Province Science and Technology Support Program (2024NSFSC0592). The funders had no role in study design, data collection and analysis, decision to publish, or preparation of the manuscript.”

4. PLOS ONE now requires that authors provide the original uncropped and unadjusted images underlying all blot or gel results reported in a submission’s figures or Supporting Information files. This policy and the journal’s other requirements for blot/gel reporting and figure preparation are described in detail at https://journals.plos.org/plosone/s/figures#loc-blot-and-gel-reporting-requirements and https://journals.plos.org/plosone/s/figures#loc-preparing-figures-from-image-files. When you submit your revised manuscript, please ensure that your figures adhere fully to these guidelines and provide the original underlying images for all blot or gel data reported in your submission. See the following link for instructions on providing the original image data: https://journals.plos.org/plosone/s/figures#loc-original-images-for-blots-and-gels. .

In your cover letter, please note whether your blot/gel image data are in Supporting Information or posted at a public data repository, provide the repository URL if relevant, and provide specific details as to which raw blot/gel images, if any, are not available. Email us at plosone@plos.org if you have any questions.

**Responses**: Thank you for bringing this policy to our attention. We confirm that our study does not include any blot- or gel-based experiments (e.g., Western blots or electrophoretic gels), and no blot/gel images are presented in either the main figures or the Supporting Information. Therefore, the requirement to provide original uncropped blot/gel images does not apply to this submission.

5. We note that your Data Availability Statement is currently as follows: [All relevant data are within the manuscript and its Supporting Information files.]

Please confirm at this time whether or not your submission contains all raw data required to replicate the results of your study. Authors must share the “minimal data set” for their submission. PLOS defines the minimal data set to consist of the data required to replicate all study findings reported in the article, as well as related metadata and methods (https://journals.plos.org/plosone/s/data-availability#loc-minimal-data-set-definition).

For example, authors should submit the following data:

- The values behind the means, standard deviations and other measures reported;

- The values used to build graphs;

- The points extracted from images for analysis.

Authors do not need to submit their entire data set if only a portion of the data was used in the reported study.

If your submission does not contain these data, please either upload them as Supporting Information files or deposit them to a stable, public repository and provide us with the relevant URLs, DOIs, or accession numbers. For a list of recommended repositories, please see https://journals.plos.org/plosone/s/recommended-repositories.

If there are ethical or legal restrictions on sharing a de-identified data set, please explain them in detail (e.g., data contain potentially sensitive information, data are owned by a third-party organization, etc.) and who has imposed them (e.g., an ethics committee). Please also provide contact information for a data access committee, ethics committee, or other institutional body to which data requests may be sent. If data are owned by a third party, please indicate how others may request data access.

**Responses**: Thank you for your careful review and valuable suggestions. We fully understand and appreciate the importance of providing the minimal data set for ensuring reproducibility and transparency. However, due to third-party agreements and institutional confidentiality requirements at the site where this study was conducted, we are unable to publicly deposit the underlying raw data in an open platform or public repository. If needed, interested editors or readers may contact the corresponding author to request access; the relevant data can be made available upon reasonable request and subject to compliance with the applicable confidentiality regulations.

In addition, we have addressed the statistical reporting request by providing the F statistics and p values for our comparisons (key comparisons in the main text, with the complete statistical outputs provided as Supplementary Data). We have also uploaded an additional Supporting Information table titled “Sequences of primers for real-time quantitative PCR”.

6. Please include a separate caption for each figure in your manuscript.

**Responses**: We thank the editor for this suggestion. Separate captions have been provided for each figure in the revised manuscript.

7. If the reviewer comments include a recommendation to cite specific previously published works, please review and evaluate these publications to determine whether they are relevant and should be cited. There is no requirement to cite these works unless the editor has indicated otherwise.

**Responses**: We have carefully reviewed and evaluated the publications suggested by the reviewers with respect to their relevance to our study. We appreciate these recommendations and have taken them into consideration when revising the manuscript.
==============================
Reviewers' comments:
Reviewer's Responses to Questions

**Comments to the Author**

Reviewer #1: This is an interesting and well-elaborated study investigating how food-based multisensory stimulation (visual, olfactory, and gustatory cues) modulates gut and brain inflammation following mTBI in rats. The work is original and methodologically solid, combining behavioral analysis, histopathology, microbiome profiling, and in vitro microglial assays. The concept — targeting the gut–brain axis (GBA) through sensory and nutritional stimulation — is novel and aligns with the growing interest in neurogastroenterology and predictive, preventive, and personalized medicine (PPPM).
However, some mechanistic links remain associative, and the translational dimension could be better developed. The manuscript would benefit from (1) clarifying causation versus correlation, (2) expanding on microbiome–brain mechanisms, including probiotic and prebiotic strategies, and (3) connecting experimental findings with clinical markers and evaluation methods of GBA dysfunction and visceral pain.
Major Comments
1. Causation versus correlation
The study convincingly shows that multisensory stimulation alters the intestinal microbiota, increases butyrate levels, and improves neurological and inflammatory outcomes.
However, causality between microbiome modulation and neuroinflammation reduction is not established. The language should be adjusted to avoid overinterpretation (“associated with” rather than “caused by”).
Recommendations:
Discuss or, if possible, perform experiments to test causality:
- Antibiotic depletion or fecal microbiota transfer (FMT) to determine if behavioral benefits depend on microbiota.
- Butyrate pathway perturbation (e.g., receptor antagonism or HDAC inhibition) to confirm mechanistic relevance.
- Time-course analysis to determine whether microbiome or SCFA changes precede neural improvements.
Include an explicit paragraph in the Discussion acknowledging these limitations and outlining future directions.

**Responses**: We appreciate this important comment and fully agree that our current data do not establish a causal relationship between microbiome modulation, butyrate, and the observed improvements in neuroinflammation and behavior. In the revised manuscript, we have carefully toned down causal language throughout the Results and Discussion sections and now consistently use association‑focused wording. The specific changes have been highlighted in the revised version of the manuscript. In addition, we have added a dedicated paragraph in the Discussion explicitly acknowledging these limitations and outlining future research directions.

2. Clarification of mechanisms and butyrate relevance
The in vitro evidence showing butyrate’s anti-inflammatory effect on microglia is valuable but needs quantitative context.
Please:
Report absolute serum butyrate concentrations (µM) and compare them to the 5 mM concentration used in vitro.
Add information about SCFA quantification (LLOQ, sample stability).
Clarify that in vitro butyrate concentrations may exceed physiological levels.

**Responses**: Thank you for this helpful suggestion. We agree that placing the in vitro butyrate exposure in quantitative physiological context is important. In the current study, butyrate was assessed in both fecal and serum samples, but the measurements were performed as relative abundance/relative levels rather than an analytically validated absolute quantification. Therefore, we are unable to report absolute serum butyrate concentrations or make a direct quantitative comparison to the 5 mM concentration used in vitro.

To address the reviewer’s concern, we have revised the Discussion to (i) explicitly state that our in vivo butyrate data are relative, (ii) acknowledge that the 5 mM in vitro concentration may exceed physiological levels, and (iii) highlight that future studies should include validated absolute SCFA quantification.

3. Microbiome characterization
Microbiota analyses are limited to the genus level and lack functional insight.
Recommendations:
Provide an ASV/OTU-level differential table (with effect size and p-value) as Supplementary Data.
Add functional predictions (PICRUSt2 or similar) to infer butyrate-synthesis capacity.
Discuss specific taxa known to produce butyrate (e.g., Ruminococcus, Roseburia, Faecalibacterium) and their potential role.

**Responses**: Thank you for your careful review and valuable suggestions regarding microbiome characterization. We agree that genus-level summaries provide limited resolution and that additional analyses could strengthen functional interpretation.

①ASV/OTU-level differential table (effect size and p-value) as Supplementary Data

We appreciate this recommendation. However, due to third‑party agreements and institutional confidentiality requirements at the site where this study was conducted, we are unable to publicly deposit the underlying sequencing-derived raw data and corresponding ASV/OTU-level differential tables as open Supporting Information. If needed, the corresponding author can provide the relevant results (including effect sizes and p-values) upon reasonable request and in accordance with the applicable confidentiality regulations.

②Functional predictions (PICRUSt2 or similar) to infer butyrate-synthesis capacity

We agree that functional prediction would be informative. However, given the limitations inherent to 16S rRNA gene sequencing and our study design, we did not perform inferential functional prediction (e.g., PICRUSt2) to avoid interpretations that are not directly supported by the data. Future work will prioritize shotgun metagenomic sequencing (and, where appropriate, metatranscriptomics) to more directly characterize microbial functional potential, with particular focus on key metabolic pathways including butyrate biosynthesis.

4. Translational implications — personalized microbiome modulation
The translational potential is strong but underdeveloped. Authors should integrate evidence on strain-specific and personalized probiotic or prebiotic use.
Add a short subsection: “Translational implications: microbiome modulation.”
Suggested discussion points:
Strain-specific effects (e.g., Lactobacillus vs. Bifidobacterium spp.) and safety aspects (adhesion, resistance, fermentation patterns).
Personalized selection of probiotics/prebiotics based on host phenotype and microbiome composition.
- References to PPPM studies and consensus papers, for example:
Gibson GR et al., Nat Rev Gastroenterol Hepatol (2017). DOI: 10.1038/nrgastro.2017.75.
Reid G et al., Benef Microbes (2017). DOI: 10.3920/BM2016.0222.Bubnov R., Spivak M. (2023). Pathophysiology-Based Individualized Use of Probiotics and Prebiotics for Metabolic Syndrome. In Microbiome in 3P Medicine Strategies (Springer).
EPMA J (2018). https://pmc.ncbi.nlm.nih.gov/articles/PMC5972142/
These sources outline mechanisms for distant-site probiotic activity (gut–liver, gut–brain) and provide a framework for personalized microbiome therapies.

**Responses**: We thank the reviewer for this constructive suggestion regarding the translational aspects of microbiome modulation. In response, we have added a dedicated subsection entitled “Translational implications” to the Discussion section. The specific additional information is as follows: In this study, multisensory stimulation was associated with alterations in gut microbiota composition, increased butyric acid levels, and reduced inflammation in the gut and brain, supporting the gut microbial–metabolic axis as a potential therapeutic target to improve cognitive function after mTBI. From a translational perspective, future strategies should move beyond non-specific “one-size-fits-all” probiotic or prebiotic supplementation and instead emphasize strain specificity and inter-individual differences. Studies have shown that different strains within the probiotic genera *Lactobacillus* and *Bifidobacterium* exhibit distinct characteristics in terms of adherence, antibiotic tolerance, gastrointestinal biofluid tolerance, and immunomodulatory activity ^[1]^. They also vary in terms of safety. Moreover, different *Lactobacillus* and *Bifidobacterium* strains have been reported to exert pronounced strain‑specific effects on intestinal barrier function, immune and inflammatory responses, and the function of distant organs via the gut–hepatic and gut–brain axes, and some strains even exert divergent biological effects among themselves ^[2,3]^. At the same time, individualized microbiome interventions are increasingly recognized as consistent with the concept of Predictive, Preventive and Personalized Medicine (PPPM) ^[4]^. For patients with mTBI and cognitive impairment, future translational research could combine systematic assessment of baseline gut microbiome composition, inflammatory status, and metabolic phenotype with targeted selection or combination of probiotics and prebiotics tailored to these characteristics, with the aim of enhancing neuroprotective, anti-inflammatory metabolic pathways.

We believe these additions strengthen the translational relevance of our manuscript and better align it with current PPPM-oriented strategies in microbiome research.

References:

[1] Bubnov, Rostyslav V et al. “Specific properties of probiotic strains: relevance and benefits for the host.” *The EPMA journal* vol. 9,2 205-223. 13 Apr. 2018.
[2] Gibson, Glenn R et al. “Expert consensus document: The International Scientific Association for Probiotics and Prebiotics (ISAPP) consensus statement on the definition and scope of prebiotics.” *Nature reviews. Gastroenterology & hepatology* vol. 14,8 (2017): 491-502.
[3] Reid, G et al. “How do probiotics and prebiotics function at distant sites?.” *Beneficial microbes* vol. 8,4 (2017): 521-533.
[4] Bubnov, R., Spivak, M. (2023). Pathophysiology-Based Individualized Use of Probiotics and Prebiotics for Metabolic Syndrome: Implementing Predictive, Preventive, and Personalized Medical Approach. In: Boyko, N., Golubnitschaja, O. (eds) Microbiome in 3P Medicine Strategies. Advances in Predictive, Preventive and Personalised Medicine, vol 16. Springer, Cham. https://doi.org/10.1007/978-3-031-19564-8_6

5. Integration of distant axes (gut–liver–brain and systemic effects)
The Discussion focuses on intestinal and brain findings but omits the gut–liver axis and systemic metabolic intermediates.
Please expand on how liver metabolism, vagal signaling, and systemic immune modulation may mediate brain protection.

**Responses**: We thank the reviewer for this important and insightful comment regarding the integration of distant axes, in particular the gut–liver–brain axis and systemic metabolic intermediates. In the revised manuscript, we have substantially expanded the Discussion to address these aspects in more detail. The specific content is presented in the revised manuscript.

6. Translational markers and clinical evaluation of the GBA
To bridge to clinical settings, authors should refer to measurable biomarkers and diagnostic tools used to evaluate GBA integrity and dysfunction.
Recommend adding:
Circulating and fecal SCFAs (butyrate, propionate, acetate).
Systemic inflammation and permeability markers (IL-6, TNF-α, CRP, LPS, zonulin).
CSF biomarkers (YKL-40, NfL, sTREM2) and autonomic indices (heart rate variability).
Visceral pain assessment methods (rectal/colonic barostat, validated visceral pain scales).
Possible myofascial and postural correlations in GBA disorders (constipation–motility–trigger point linkage, as in  [https://onlinelibrary.wiley.com/doi/10.1111/nmo.13671#nmo13671-sec-0439-title]).
Consider the role of central sensitization and CSF flow changes (e.g., Effects on Spinal Fluid Dynamics and Gut-Brain Axis Modulation in Migraine Patients with Central Sensitization Cephalalgia 2024, https://doi.org/10.1177/03331024241280496#sec-206).
These perspectives make the work more relevant to clinical neurology, gastroenterology, and rehabilitation.

**Responses**: We thank the reviewer for this valuable suggestion. In response to the comment on “Translational markers and clinical evaluation of the GBA”, we did not create a separate section, but instead integrated the relevant content into the existing Discussion subsection entitled “Translational implications”. The added text is as follows: On this basis, objective, axis-specific assessments of the gut–brain and gut–liver–brain axes will also be important in future translational research. Clinically, on the one hand, short-chain fatty acid profiles (especially butyric acid, propionic acid, and acetic acid) in feces and blood, as well as systemic inflammation and barrier function indices (e.g., IL‑6, TNF‑α, C‑reactive protein [CRP], LPS, and zonulin) that parallel the findings of the present study, could be used to characterize changes in the activity and permeability of the gut microbial–metabolic axis. In parallel, cerebrospinal fluid markers of inflammation and neurological injury (e.g., YKL‑40, neurofilament light chain [NfL], soluble TREM2 [sTREM2]), autonomic function indicators (e.g., heart rate variability), and visceral nociceptive assessments incorporating myofascial- and posture-related phenotypes could be integrated into a multimodal evaluation framework for patients with mTBI to quantify gut–brain and gut–liver–brain axis dysfunction and its relationship to central sensitization ^[1-3]^. These multimodal markers may be combined with individualized microbiome interventions for patient stratification, efficacy monitoring, and regimen optimization in future clinical studies.

References:

[1] Hampel, Harald et al. “Alzheimer's disease biomarker-guided diagnostic workflow using the added value of six combined cerebrospinal fluid candidates: Aβ_1-42_, total-tau, phosphorylated-tau, NFL, neurogranin, and YKL-40.” *Alzheimer's & dementia : the journal of the Alzheimer's Association* vol. 14,4 (2018): 492-501.
[2] Suárez-Calvet, Marc et al. “sTREM2 cerebrospinal fluid levels are a potential biomarker for microglia activity in early-stage Alzheimer's disease and associate with neuronal injury markers.” *EMBO molecular medicine* vol. 8,5 466-76. 2 May. 2016.
[3] BUBNOV R, KALIKA L, SPIVAK M. Constipation and gut motility is associated with posture instability: Role of muscle trigger points and potential to treat by targeted dry needling [J]. Neurogastroenterology and Motility, 2019, 31: 151.

Minor Comments
①Revise figures to show time-course trends of SCFAs and inflammatory markers alongside behavioral recovery.
②Report sample sizes and power calculation for behavioral and molecular endpoints.
③Provide details of the multisensory environment (odorant composition, decibel level, exposure duration) to ensure reproducibility.
④Deposit raw sequencing data (FASTQ) and GC–MS files in a public repository with accession numbers.
⑥Ensure consistent terminology: “gut–brain axis” (GBA) throughout.

**Responses**: Thank you for these helpful suggestions. We have revised the manuscript where feasible, and we also clarify below the items that cannot be fully addressed due to study design and/or data-sharing restrictions.

① Revise figures to show time-course trends of SCFAs and inflammatory markers alongside behavioral recovery.

We agree that time-course visualization would be valuable. However, in this study SCFAs were measured at a single post-injury time point (day 6) rather than longitudinally; therefore, we cannot present SCFA time-course trends based on the current dataset. Future studies with repeated sampling across multiple time points will be needed to characterize the temporal dynamics of SCFAs and inflammatory markers alongside behavioral recovery.

② Report sample sizes and power calculation for behavioral and molecular endpoints.

We have revised the manuscript to clearly report sample sizes (n) for all behavioral and molecular endpoints (in the Methods and figure legends). We also added a description of the a priori sample-size estimation using G*Power 3.1: the analysis indicated that 6 animals per group would provide 80% power at a two-sided α = 0.05^[1]^. To improve robustness while maintaining feasibility and ethical use of animals, we increased the group size to 8 animals.

③ Provide details of the multisensory environment (odorant composition, decibel level, exposure duration) to ensure reproducibility.

We have added further methodological details to improve reproducibility, including the odorant composition, sound level (dB), and exposure duration of the multisensory environment.

④ Deposit raw sequencing data (FASTQ) and GC–MS files in a public repository with accession numbers.

We understand the importance of public deposition of raw data. However, due to third-party agreements and institutional confidentiality requirements at the site where this study was conducted, we are unable to publicly deposit the raw 16S rRNA sequencing FASTQ files and GC–MS raw files in an open repository. If needed, the corresponding author can facilitate access to the relevant raw data upon reasonable request, subject to compliance with the applicable confidentiality regulations and any required approvals. We have updated the Data Availability information accordingly.

⑥ Ensure consistent terminology: “gut–brain axis” (GBA) throughout.

We have carefully checked the revised manuscript and standardized the terminology to use “gut–brain axis (GBA)” consistently throughout.

References:

[1] Festing, Michael Fw. “On determining sample size in experiments involving laboratory animals.” *Laboratory animals* vol. 52,4 (2018): 341-350.

==============================

Reviewer #2: This manuscript presents a promising approach to treating cognitive impairment after mild traumatic brain injury using food-based multisensory stimulation. The in vivo work is generally well-executed with comprehensive behavioral, microbiome, and histological analyses that convincingly demonstrate therapeutic benefits. However, the study suffers from several critical weaknesses.

1. The planning of the cognitive experiments (Morris water and nort) in the early post-TBI days may be confounded by sensorimotor impairments and neurological severity, making it difficult to accurately assess cognitive function. The authors should clarify how they accounted for this potential confound.

**Responses**: We appreciate this important comment. We agree that early post‑TBI sensorimotor deficits can potentially confound cognitive readouts. In our study, all rats were pretrained on the Morris water maze before surgery to ensure comparable baseline task acquisition, and we monitored neurological severity daily using the mNSS. During post‑injury testing, we recorded swimming speed in the MWM and total exploration time in the NOR and found no significant group differences in these measures, indicating that gross motor or motivational impairments were unlikely to account for the observed cognitive differences. These points have now been explicitly clarified in the Methods section (Morris water maze, Novel object recognition, and mNSS).

2. The introduction should be revised to clearly and concisely address the gut–brain connection in mTBI, the associated cognitive impairments, and the rationale for exploring food-based interventions in TBI (PMID: 36481824, 39751900).

**Responses**: We thank the reviewer for this insightful comment. In the revised Introduction, we have now (i) more clearly described the gut–brain axis in the context of mTBI and its contribution to long-term cognitive impairment, (ii) summarized recent evidence showing that gut-directed, food-related interventions such as quercetin and chitosan lactate can ameliorate rmTBI-induced neuroinflammation and cognitive deficits (PMID: 36481824, 39751900), and (iii) more explicitly articulated the rationale for exploring food-based multisensory stimulation as a non-pharmacological strategy to modulate the microbiota–gut–brain axis after mTBI.

3. What was the exact timeline for all the experiments in sequence? A study design figure depicting the time should be made.

**Responses**: We appreciate the reviewer’s suggestion. We have now added a study design figure that clearly presents the exact experimental timeline in chronological order, including all behavioral assessments. This new figure has been included as Figure 1 in the revised manuscript. Importantly, Figure 1 simultaneously depicts the experimental workflow and the intervention setup, i.e., the food-based multisensory stimulation apparatus, to provide a complete overview of the study design and procedures.

4. For the estimation of NSE and S-100β, the same animals were used for different timepoints or different animals, and this should be clearly stated in the methods section.

**Responses**: We appreciate the reviewer’s comment. In this study, venous blood was collected longitudinally from the same animals at all time points (baseline and post-injury days 2, 4, and 6). This information has now been explicitly clarified in the Methods section.

5. Interventions commenced immediately post-injury and continued for seven days, operating around the clock. But there is no rationale for why 7 days were chosen. No citation of previous multisensory stimulation studies using this duration. No pilot data mentioned. No dose-finding or optimization experiments. The choice appears arbitrary.

**Responses**: We thank the reviewer for raising this important point. In the revised manuscript, we have added the biological rationale for choosing a 7‑day intervention period and have clarified this explicitly in the Methods section.

Specifically, our intervention was designed to cover the acute and early subacute phase after mTBI, which is considered a critical time window during which neuroinflammatory responses and gut–brain axis alterations are most pronounced and most likely to shape subsequent disease progression. A recent mouse CCI study demonstrated that intestinal permeability shows a transient increase as early as ~4 h after TBI, while marked changes in medial colonic goblet cell density, colonic hypoxia, and the abundance of key bacterial taxa such as *Akkermansia muciniphila* occur between 1 and 3 days post-injury; thereafter, although no overt intestinal morphological damage is observed, the gut micro-ecosystem and related parameters enter a relatively slower phase of remodeling ^[1]^. These findings suggest that the first week after injury represents a critical period during which gut barrier function and microbiota composition are highly plastic and potentially most amenable to intervention.

In addition, although studies specifically focusing on food-based multisensory stimulation are still limited, a previous study investigating whether quercetin—a flavonoid with anti-inflammatory and antioxidant properties—could act as a “prebiotic” to improve the disrupted gut microbiota–brain axis in a repeated mild TBI (rmTBI) mouse model also employed a 7‑day intervention regimen ^[2]^.

Taken together, these data support our decision to initiate food-related multisensory stimulation immediately after injury and to maintain it for 7 consecutive days, in order to encompass this acute–early subacute pathological window and thereby evaluate its early modulatory effects on gut–brain axis dysregulation and cognitive function.

References:

[1] DeSana, Anthony J et al. “Acute gastrointestinal permeability after traumatic brain injury in mice precedes a bloom in Akkermansia muciniphila supported by intestinal hypoxia.” *Scientific reports* vol. 14,1 2990. 5 Feb. 2024,.

[2] Balasubramanian, Ramya et al. “Involvement of Microbiome Gut-Brain Axis in Neuroprotective Effect of Quercetin in Mouse Model of Repeated Mild Traumatic Brain Injury.” *Neuromolecular medicine* vol. 25,2 (2023): 242-254.

6. Also 7 days captures only the acute phase, Gut microbiome remodeling typically takes 2-4 weeks to stabilize

**Responses**: We thank the reviewer for this comment and agree that remodeling of the gut microbiota may extend over several weeks, with community composition often requiring 2–4 weeks or longer to stabilize. However, in the present study, our 7‑day intervention was intentionally designed to target the period during which neuroinflammation, gut barrier function, and microbiota composition undergo the most rapid changes and may be most amenable to modulation. Therefore, we chose a 7‑day continuous intervention to encompass this early phase rather than the entire stabilization period. We acknowledge that our design does not address whether a longer intervention would produce greater or more sustained effects. We have added this point as a limitation in the Discussion and noted that future studies should compare different intervention durations to determine the optimal length of food‑based multisensory stimulation after mTBI.

7. The idea of this work revolves around the food based multisensory stimulation. So, was the amount of food consumed by each animal noted throughout the study plan, and what about the weight of the animals data?

**Responses**: We thank the reviewer for this important question. In the present study, we did not systematically record individual food intake or body weight over the course of the experiment. All animals were housed under identical conditions with ad libitum access to standard chow and water, and no overt signs of altered general condition (e.g., poor grooming, dehydration, or obvious weight loss) were noted during routine daily monitoring by the animal care staff and investigators.

Our primary focus in this work was to examine the effects of food‑based multisensory stimulation on behavior and gut–brain axis–related outcomes, rather than on nutritional or metabolic parameters per se. Moreover, the paradigm involved brief, experimenter‑controlled exposure to food‑related sensory cues rather than prolonged access to additional or highly palatable food, which reduces the likelihood of large group differences in overall caloric intake.

For these reasons, although we cannot provide quantitative intake or body weight data, we do not expect major systematic differences between groups that would account for the observed behavioral and gut–brain axis effects. Nonetheless, we agree that future studies using similar paradigms should incorporate systematic monitoring of food consumption and body weight to more precisely characterize potential metabolic or nutritional contributions.

8. In the relative mRNA expression results, what is ACTB?

**Responses**: We apologize for the lack of clarity. ACTB refers to β‑actin (beta‑actin), which was used as the housekeeping/reference gene for normalization of target mRNA expression in our qPCR analyses. We have now clarified this in the Methods by explicitly stating “ACTB (β‑actin)” as the internal control.

9. The Nissl-positive area has significantly improved in the food-based multisensory group in the CTX and CA3 regions, but not in the CA1 region. What could be the reason?

**Responses**: We appreciate the reviewer’s thoughtful comment. In our study, food‑based multisensory stimulation attenuated Nissl‑defined neuronal loss in the peri‑lesional cortex (CTX) and hippocampal CA3, but we did not observe a comparable rescue in the CA1 region. There are several possible explanations.

The differential protective effects observed in various subregions of the hippocampus—that is, neurons in area CA3 and the cortex surrounding the injury were relatively better protected, whereas no significant improvement was seen in area CA1—suggest that the effects of multisensory stimulation and intestinal metabolites may be brain-region specific. Previous studies have shown that CA1 pyramidal neurons are highly sensitive to ischemia, excitotoxicity, and a variety of secondary injury cascades, whereas CA3 and certain cortical regions have relatively greater potential for structural plasticity and compensatory remodeling ^[1-2]^. In addition, the role of gut-derived SCFAs in the brain may also exhibit regional differences. It has been suggested that SCFA receptors (e.g., GPR41, GPR43), as well as transporter proteins mediating SCFA uptake, are not uniformly distributed in expression across brain regions and cell types ^[3-4]^. If CA3 neurons or neurons, astrocytes, and microglia in the cortex are more sensitive to SCFA receptor signaling, then these regions may be more susceptible to anti-inflammatory and neuroprotective effects in the context of elevated systemic levels of butyric acid induced by multisensory stimulation, whereas CA1 may be relatively “underresponsive.” Future studies are warranted to further validate these findings at the tissue and cellular levels.

This paragraph has been incorporated into the Discussion section of the revised manuscript.

References:

[1] Merino-Serrais, Paula et al. “Structural changes of CA1 pyramidal neurons after stroke in the contralesional hippocampus.” *Brain pathology (Zurich, Switzerland)* vol. 34,3 (2024): e13222.
[2] Stanika, Ruslan I et al. “Differential NMDA receptor-dependent calcium loading and mitochondrial dysfunction in CA1 vs. CA3 hippocampal neurons.” *Neurobiology of disease* vol. 37,2 (2010): 403-11.
[3] Silva, Ygor Parladore et al. “The Role of Short-Chain Fatty Acids From Gut Microbiota in Gut-Brain Communication.” *Frontiers in endocrinology* vol. 11 25. 31 Jan. 2020.
[4] Lee, Do-Hyung et al. “GPR41 and GPR43: From development to metabolic regulation.” *Biomedicine & pharmacotherapy = Biomedecine & pharmacotherapie* vol. 175 (2024): 116735.

10. Was an established protocol followed for conducting the behavioral experiments? The method used for calculating the Discrimination Index appears to be adapted from previously published protocols, and therefore, relevant literature should be referenced (e.g., PMID: 38423243).

**Responses**: We thank the reviewer for this helpful comment. Yes, our behavioral experiments were conducted according to established and widely used protocols. Specifically, the method for calculating the Discrimination Index in the novel object recognition test was based on standard procedures reported in previous studies. In the revised manuscript, we have clarified this point in the Methods section and added the recommended reference accordingly.

11. Please clarify how the food items (pet biscuits, chicken jerky) were selected and provide their nutritional composition. Also specify whether the scents were standardized, the concentrations used, and the volume/intensity of the chewing sounds. Indicate whether rats had physical access to the food or only sensory exposure. A brief, detailed protocol describing all stimulus parameters (duration, concentration, intensity) and a simple methods figure of the setup would improve clarity.

**Responses**: We thank the reviewer for these constructive suggestions. In the revised Methods, we now provide a more detailed description of the food-based multisensory stimulation protocol. Our intervention was derived from an integrated audio-visual-olfactory virtual reality sham-feeding device previously developed by our team for clinical application; this device simulates the oral feeding state and stimulates brain neurons via integrated audio-visual-olfactory sensory cues to induce related physiological responses and functional expression, thereby compensating for the limitations of tube feeding or enteral nutrition delivery by oral administration alone ^[1]^。Specifically, we clarify that pet biscuits and chicken jerky were selected based on pilot tests of rats’ preference, and we report their nutritional composition according to the manufacturer’s specifications. We also specify that a sweet-type scent was used and diluted 1:50 (v/v) before being applied to cotton balls inside the stimulus box. The chewing-sound recordings were played at an intensity of approximately 50–60 dB at the level of the animal. We further emphasize that rats were exposed only to the sensory properties of the food and had no physical access to the food items throughout the experiment. Finally, we have added a brief, detailed description of all stimulus parameters (duration, concentration, intensity) and included a schematic figure illustrating the setup (Figure 1) to improve clarity.

Reference:

[1] Li K, Yuan X, Hu Y, Zhang W, Chen Y, Hong R, Yang J: [An Integrated Audio-Visual-Olfactory Virtual Reality False Feeding Device: Research, Development, and Design]. Sichuan Da Xue Xue Bao Yi Xue Ban 2023, 54:792-797.

12. While the in vitro data (Fig. 4O) shows anti-inflammatory effects of butyrate, it does not establish that butyrate mediates the in vivo cognitive benefits of sensory stimulation; the authors should either include a butyrate-only or butyrate-blockade group, or clearly acknowledge this limitation in the discussion.

**Responses**: We thank the reviewer for this important comment. We fully agree that our in vitro findings, which demonstrate anti‑inflammatory effects of butyrate, do not by themselves prove that butyrate is the causal mediator of the in vivo cognitive benefits observed after food‑based multisensory stimulation. In the revised manuscript, we now explicitly acknowledge this limitation in the Discussion and emphasize that future studies are needed to investigate this mechanism more directly, for example by determining whether blockade of butyrate signaling attenuates the neuroprotective and cognitive effects of the sensory intervention.

13. With numerous outcome measures across multiple timepoints and tissue types, correction for multiple comparisons should be applied or justified if not applied

**Responses**: We thank the reviewer for raising this important statistical issue. In the present study, we did apply corrections for multiple comparisons within each set of related tests. We recognize that this was not described with sufficient clarity in the original manuscript. In the revised version, we have expanded the Statistical Analysis section to clearly specify the multiple‑comparison procedures used.

14. A sensory stimulation + sham injury group would help determine if the intervention affects healthy animals or is specific to mTBI pathology. Either add this control group or explicitly discuss this limitation and its implications for interpreting the specificity of the intervention.

**Responses**: We appreciate the reviewer’s insightful suggestion. We agree that a “sensory stimulation + sham injury” group would be informative for determining whether the effects of food‑based multisensory stimulation also occur in healthy animals. However, the present study was specifically designed and powered to address a disease‑oriented question, namely whether this intervention ameliorates mTBI‑related neuropathology and cognitive deficits compared with injured animals without stimulation. Within this scope, we consider the current control groups sufficient to support our main conclusions, and we view the systematic evaluation of sensory stimulation in sham‑injured animals as an important direction for future, separately designed studies.

15. The correlation between specific taxa and SCFAs is interesting, but doesn't prove causality. Consider softening language.

**Responses**: We appreciate the reviewer’s helpful comment. We fully agree that the observed correlations between specific bacterial taxa and SCFA levels do not establish causality. In the revised manuscript, we have softened the wording accordingly. We also clearly state in the Discussion that these correlative data do not prove causality and that mechanistic studies will be required to determine causal links.

16. The differential effects across brain regions (cortex and CA3 protected, CA1 not protected) are intriguing but underexplored. Discuss potential mechanisms for regional specificity. Could it relate to differential expression of SCFA receptors (GPR41, GPR43) or regional vulnerability to the injury model?

**Responses**: We thank the reviewer for this insightful comment. In the revised Discussion, we have now explicitly addressed potential mechanisms underlying the observed regional specificity. The newly added content is as follows: The differential protective effects observed in various subregions of the hippocampus—that is, neurons in area CA3 and the cortex surrounding the injury were relatively better protected, whereas no significant improvement was seen in area CA1—suggest that the effects of multisensory stimulation and intestinal metabolites may be brain-region specific. Previous studies have shown that CA1 pyramidal neurons are highly sensitive to ischemia, excitotoxicity, and a variety of secondary injury cascades, whereas CA3 and certain cortical regions have relatively greater potential for structural plasticity and compensatory remodeling ^[1-2]^. In addition, the role of gut-derived SCFAs in the brain may also exhibit regional differences. It has been suggested that SCFA receptors (e.g., GPR41, GPR43), as well as transporter proteins mediating SCFA uptake, are not uniformly distributed in expression across brain regions and cell types ^[3-4]^. If CA3 neurons or neurons, astrocytes, and microglia in the cortex are more sensitive to SCFA receptor signaling, then these regions may be more susceptible to anti-inflammatory and neuroprotective effects in the context of elevated systemic levels of butyric acid induced by multisensory stimulation, whereas CA1 may be relatively “underresponsive.” Future studies are warranted to further validate these findings at the tissue and cellular levels.This paragraph has been incorporated into the Discussion section of the revised manuscript.

References:

[1] Merino-Serrais, Paula et al. “Structural changes of CA1 pyramidal neurons after stroke in the contralesional hippocampus.” *Brain pathology (Zurich, Switzerland)* vol. 34,3 (2024): e13222.

[2] Stanika, Ruslan I et al. “Differential NMDA receptor-dependent calcium loading and mitochondrial dysfunction in CA1 vs. CA3 hippocampal neurons.” *Neurobiology of disease* vol. 37,2 (2010): 403-11.

[3] Silva, Ygor Parladore et al. “The Role of Short-Chain Fatty Acids From Gut Microbiota in Gut-Brain Communication.” *Frontiers in endocrinology* vol. 11 25. 31 Jan. 2020.

[4] Lee, Do-Hyung et al. “GPR41 and GPR43: From development to metabolic regulation.” *Biomedicine & pharmacotherapy = Biomedecine & pharmacotherapie* vol. 175 (2024): 116735.

17. Abstract: "may be associated" and "possibly promoting" are appropriately cautious but could be stronger given your data. Consider removing some hedging language where data directly support conclusions

**Responses**: We thank the reviewer for this helpful suggestion. In the revised abstract, we have reduced unnecessary hedging where the data provide direct support. We have retained cautious wording only where causal relationships cannot be firmly established.

18. Methods section numbering is inconsistent (2.4 appears twice)

**Responses**: We thank the reviewer for pointing out this formatting error. In the revised manuscript, we have carefully checked and corrected the numbering of all subsections in the Methods section, ensuring that each subsection now has a unique and consistent number.

19. What was the microglial cell source, primary cells? (from rats? mice?) or Cell line? (BV2? N9? …..)

**Responses**: We thank the reviewer for this question. In the in vitro experiments, we used the murine microglial cell line BV2 rather than primary microglia. We have now clarified this information in the Methods section.

20. Consider discussing the acute (7-day) vs. chronic nature of the intervention and whether effects would persist after cessation

**Responses**: We appreciate this important suggestion. In the revised Discussion, we now address the acute (7‑day) nature of our intervention and its implications for long‑term effects. Specifically, we note that the 7‑day protocol primarily targets the acute to early subacute phase after mTBI, whereas stable remodeling of the gut microbiota may require a longer time frame. We also acknowledge that prolonged or continuous multisensory stimulation might confer additional or more sustained benefits. These points have been added to the Discussion as a limitation of the present study.

21. In Figure 3, HE staining images need scale bars. Quantification of villus height, crypt depth, or villi to crypt ratio and goblet cell density would strengthen conclusions.

**Responses**: We appreciate the reviewer’s helpful comments. We have now added scale bars to the H&E staining images in Figure 3 to improve clarity. Regarding the suggested quantitative analyses of villus height, crypt depth, villus‑to‑crypt ratio, and goblet cell density, we fully agree that such measurements would further strengthen the conclusions. However, these parameters were not predefined in our original analysis plan, and the available images and sections were not collected or digitized in a way that allows reliable retrospective morphometric quantification. To avoid introducing potentially inaccurate measurements, we have therefore limited our intestinal histology assessment to the qualitative description presented in Figure 4.

22. Figure 4 is dense with information, consider splitting it into two figures. The correlation heatmap needs clearer labeling

**Responses:** We thank the reviewer for this helpful suggestion. In the revised manuscript, we have split the original Figure 4 into two figures and improved the correlation heatmap by clarifying the axis labels and legend to enhance readability.

23. Could isoflurane exposure affect gut microbiota? Did sham animals receive equivalent anesthesia duration?

**Responses**: We appreciate the reviewer’s thoughtful comment. In our study, sham animals were exposed to isoflurane anesthesia for an equivalent duration and under the same conditions as mTBI animals, but without induction of injury, in order to control for potential anesthesia-related effects on the gut microbiota and other outcomes. We have now clarified this point in the Methods section of the revised manuscript.

24. How were fecal samples collected to avoid environmental contamination? Were samples from individual pellets or the cage floor?

**Responses**: We thank the reviewer for raising this question. To minimize environmental contamination, freshly excreted fecal pellets from each rat were immediately collected using sterile forceps. The samples were then promptly snap-frozen in liquid nitrogen and stored at −80 °C. This content has been described in the revised Methods section.

25. Only male rats were used. Justify this choice and discuss how results might differ in females, given known sex differences in TBI outcomes and microbiome composition.

**Responses**: We appreciate this important comment. In this study, we primarily used male rats to reduce variability associated with the estrous cycle. We acknowledge that this design limits the generalizability of our findings to female animals. In the revised Discussion, we explicitly note this limitation and emphasize that including female or both sexes will be an important direction for future research. In addition, we have specified “male rats” in the title and clarified the rationale for using male animals in the Discussion section.

26. Were animals group housed or single housed? This significantly affects the microbiome.

**Responses**: We thank the reviewer for this important comment. In our study, rats were group housed (4 animals per cage) under standard SPF conditions. We have now specified the housing conditions in the Methods section of the revised manuscript.

27. The sample size for each experiment and each group should be indicated in the figure legends to ensure transparency, rigor, and reproducibility of the data.

**Responses**: We thank the reviewer for this helpful suggestion. In the revised manuscript, we have added the sample size (n) for each group and each experiment in all figure legends to improve transparency and reproducibility.

28. Consider revisiting the manuscript to avoid typos and grammatical errors.

**Responses**: We thank the reviewer for this remark. We have carefully re-edited the entire manuscript to correct typographical and grammatical errors and to improve the overall clarity and readability of the text.
