# Supplementary material for: Food-based multisensory stimulation ameliorates cognitive impairment after mild traumatic brain injury in male rats by modulating intestinal and brain inflammation
Source: PLoS One. 2026 Feb 27;21(2):e0343814. doi: 10.1371/journal.pone.0343814 (PMC12948070; doi:10.1371/journal.pone.0343814)
Supplement: Attachment — Submitted filename: Responses.docx [file pone.0343814.s006.docx]

**Responce to reviewer**

Thank you very much for sparing your precious time in reviewing our paper, and for your reviewer comments. We appreciate the time and effort that you and the reviewer dedicated to providing feedback on our manuscript and are grateful for the insightful comments on and valuable improvements to our paper. We have incorporated most of the suggestions made by the reviewer. Those changes are highlighted within the manuscript. Please see below, in red, for a point-by-point response to the reviewers’ comments and concerns. We hope the revised version is now suitable for publication and look forward to hearing from you in due course.

1. I still don't see the filled out ARRIVE guidelines sheet. I see only the supplementary figures and the raw data as supplementary material.

**Responses**: Thank you for your comment. The completed ARRIVE guidelines checklist has now been uploaded as a supplementary file.

2. In regards to the request: "In the results section, for comparisons, please list the F statistic for the group, and please list any p value for any comparison that you make." You responded regarding concerns for space/word limitation. As PLOSone is only online, there is no specific word limit. Again, please list the F statistic for the group, and please list any p value for any comparison that you make. Thank you.

**Response**: Thank you for the clarification and for reiterating this important reporting requirement. We apologize for any confusion caused by our previous response regarding space or word limitations. In accordance with the editor’s request, we have now revised the Results section to explicitly report the F statistic for each group comparison and to list the corresponding P value for every comparison made, including both statistically significant and non‑significant results. In addition, to ensure complete transparency and ease of review, we have compiled all reported group comparisons, together with their corresponding F statistics and P values, into a supplementary table, which has been uploaded as a Supplementary File. Thank you for your guidance, which has helped strengthen the presentation of our results.

3. In your figures, you compare the sham group to the TBI-control group, and you compare the TBI-control group to the TBI+multisensory stim group. Please also show the comparisons between the sham group and the TBI+multisensory stim group. When using multiple groups for an experiment, you cannot arbitrarily decide which groups to use for multiple comparisons. Please include this analysis as requested.

**Response**: Thank you for this important comment. We agree that, when multiple experimental groups are included, all relevant pairwise comparisons should be reported, and that it is not appropriate to selectively present only a subset of group comparisons. In response to the editor’s request, we have now performed and included the additional pairwise comparisons between the sham group and the TBI + multisensory stimulation group for all relevant experiments.

Specifically, we have:

- Added the sham vs. TBI + multisensory stimulation comparisons to the statistical analyses, with the corresponding F statistics and P values reported.
- Updated the figure panels to display these additional comparisons, including appropriate statistical annotations.
- Provided the full results of all pairwise group comparisons as supplementary data, which have been uploaded as a Supplementary File and referenced in the revised manuscript.

These revisions ensure that all group comparisons are presented transparently and consistently, in accordance with the editor’s guidance. We thank the editor for highlighting this issue, which has helped improve the rigor and completeness of our statistical analyses.
